# Membrane Radiolabelling of Exosomes for Comparative Biodistribution Analysis in Immunocompetent and Immunodeficient Mice - A Novel and Universal Approach

**DOI:** 10.7150/thno.27891

**Published:** 2019-02-28

**Authors:** Farid N. Faruqu, Julie Tzu-Wen Wang, Lizhou Xu, Luke McNickle, Eden Ming-Yiu Chong, Adam Walters, Mark Gurney, Aled Clayton, Lesley A. Smyth, Robert Hider, Jane Sosabowski, Khuloud T. Al-Jamal

**Affiliations:** 1 Institute of Pharmaceutical Science, Faculty of Life Sciences & Medicine, King's College London, Franklin-Wilkins Building, 150 Stamford Street, London SE1 9NH, United Kingdom; 2 School of Medicine, Tenovus Building, University Hospital of Wales, Heath Park, Cardiff, CF14 4XN; 3 School of Health Sport and Bioscience, University of East London, Water Lane, London E15 4LZ; 4 Centre for Molecular Oncology, Barts Cancer Institute, Queen Mary University of London, Charterhouse Square, London EC1M 6BQ

**Keywords:** exosomes, drug delivery, radiolabelling, biodistribution

## Abstract

Extracellular vesicles, in particular exosomes, have recently gained interest as novel drug delivery vectors due to their biological origin and inherent intercellular biomolecule delivery capability. An in-depth knowledge of their *in vivo* biodistribution is therefore essential. This work aimed to develop a novel, reliable and universal method to radiolabel exosomes to study their *in vivo* biodistribution.

**Methods**: Melanoma (B16F10) cells were cultured in bioreactor flasks to increase exosome yield. B16F10-derived exosomes (Exo_B16_) were isolated using ultracentrifugation onto a single sucrose cushion, and were characterised for size, yield, purity, exosomal markers and morphology using nanoparticle tracking analysis (NTA), protein measurements, flow cytometry and electron microscopy. Exo_B16_ were radiolabelled using 2 different approaches - intraluminal labelling (entrapment of ^111^Indium *via* tropolone shuttling); and membrane labelling (chelation of ^111^Indium *via* covalently attached bifunctional chelator DTPA-anhydride). Labelling efficiency and stability was assessed using gel filtration and thin layer chromatography. Melanoma-bearing immunocompetent (C57BL/6) and immunodeficient (NSG) mice were injected intravenously with radiolabelled Exo_B16_ (1x10^11^ particles/mouse) followed by metabolic cages study, whole body SPECT-CT imaging and *ex vivo* gamma counting at 1, 4 and 24 h post-injection.

**Results**: Membrane-labelled Exo_B16_ showed superior radiolabelling efficiency and radiochemical stability (19.2 ± 4.53 % and 80.4 ± 1.6 % respectively) compared to the intraluminal-labelled exosomes (4.73 ± 0.39 % and 14.21 ± 2.76 % respectively). Using the membrane-labelling approach, the *in vivo* biodistribution of Exo_B16_ in melanoma-bearing C57Bl/6 mice was carried out, and was found to accumulate primarily in the liver and spleen (~56% and ~38% ID/gT respectively), followed by the kidneys (~3% ID/gT). Exo_B16_ showed minimal tumour i.e. self-tissue accumulation (~0.7% ID/gT). The membrane-labelling approach was also used to study Exo_B16_ biodistribution in melanoma-bearing immunocompromised (NSG) mice, to compare with that in the immunocompetent C57Bl/6 mice. Similar biodistribution profile was observed in both C57BL/6 and NSG mice, where prominent accumulation was seen in liver and spleen, apart from the significantly lower tumour accumulation observed in the NSG mice (~0.3% ID/gT).

**Conclusion**: Membrane radiolabelling of exosomes is a reliable approach that allows for accurate live imaging and quantitative biodistribution studies to be performed on potentially all exosome types without engineering parent cells.

## Introduction

Exosomes are a subtype of extracellular vesicles (EV) ranging from 50-200 nm in diameter, secreted by various cell types such as dendritic cells 1, macrophages 2, cancer cells 3-6 and mesenchymal stem cells 7. Exosomes have also been shown to be present in various physiological fluids 8-11. The combination of the inherent ability of exosomes to carry various biomolecules (e.g. RNA and proteins) 12-14 and the effective delivery of these biomolecules into recipient cells 15-17 attracted interest for their potential as nano-scale drug delivery vectors for a multitude of therapeutic agents. Small molecule- drugs such as doxorubicin 18-20, paclitaxel 21, imatinib 22, curcumin 23-25, acridine orange 26 and anthocyanidin 27 have been demonstrated to be successfully loaded into exosomes and delivered to target cells. Nucleic acids such as siRNA 28, 29 and microRNA 30 have also been successfully loaded into exosomes *via* electroporation and delivered to target cells. Exosomes can also be engineered for targeted delivery, mostly by means of expressing the targeting moiety as a fusion protein with transmembrane proteins on the exosomes 15. The RVG peptide-Lamp2b fusion protein was the first to be demonstrated to target exosomes across the blood- brain barrier (BBB) for brain delivery 31. Exosomes bearing the GE11 peptide-PDGFR fusion protein were shown to target EGFR-overexpressing breast cancer cell lines 32. Interestingly, non-targeted exosomes have been reported to home to their tissue or cell of origin 33, suggesting that exosomes might have inherent targeting ability without requiring any engineering.

Given the huge interest and potential in developing exosomes as drug delivery vectors, it is essential to understand their *in vivo* biodistribution. Several studies have been conducted to analyse this, mostly involving labelling exosome with fluorescent probes to track them *in vivo via* qualitative live imaging or quantitative *ex vivo* organ analysis. The major drawback of this technique is auto-fluorescence and tissue penetration depth during live imaging, even when using near infrared (NIR) fluorescent probes, therefore requiring the animals to be culled and organs excised for *ex vivo* imaging for more reliable results 19, 32-34. This makes optical imaging limited to end-point analysis and not amenable to longitudinal studies or those that involve multiple dosing on the same animal. *Ex vivo* organ analysis using this modality also harbours substantial inaccuracies, as the fluorescence from the excised organs are detected in a 2D-manner. Combined with the limited tissue penetration depth of fluorescent probes, this results in partial loss of signals and therefore rendering the biodistribution analysis only semi-quantitative 19, 33, 34. Labelling using lipophilic dyes such as PKH26 or DiR have been reported to suffer from non-specific transfer of the dye between membranes, which heavily and adversely influenced the accuracy of the results obtained in the studies carried out 35-37. The long half-life of these lipophilic dyes adds to the drawback described above, where it is not possible to distinguish whether the signal is coming from the labelled body of interest or free dye transferred to another membrane. Therefore, the reliability and accuracy of organ biodistribution of exosomes labelled using such dyes is questionable.

Other modalities such as bioluminescence has also been explored, whereby the exosomes were engineered to express luciferase on their surface, effectively creating bioluminescent exosomes upon introduction of its substrate. This modality eliminates the problem of auto-fluorescence but requires genetic modification of the parent cells from which the exosomes originate. This can be challenging to perform on primary cells and is not possible for exosomes isolated from physiological fluids 38, 39.

Labelling the exosomes with radioactive isotopes for tracking them *in vivo* is a more robust modality for evaluating both qualitative and quantitative exosome biodistribution *via* live SPECT or PET imaging and *ex vivo* organ analysis, as it does not have the limitations associated with the modalities described above. However, only a limited number of studies have been carried out, and in these cases the radiolabelling techniques suffer from serious limitation. In one study, the exosomes were engineered to express streptavidin as a fusion protein on their membrane, and radiolabelling was achieved when incubated with ^125^I-tagged biotin 40. Again, this method requires genetic modification of the parent cells and is therefore not applicable to all types of exosomes. Another study carried out radiolabelling by entrapping the ^99m^Tc-HMPAO complex within the lumen of exosome nanomimetics (cells extruded to become vesicles of similar size to exosomes), *via* an in-situ glutathione-dependent reduction of the HMPAO chelator 41. The success of application of this method to actual exosomes is uncertain as glutathione, the key molecule for this radiolabelling method, is only found in very low amounts in exosomes 42. A similar method of entrapping the radioisotope for labelling exosomes is used in a different study, where the ^111^In-oxine complex was used to shuttle the ^111^In^3+^ ions inside the exosomal lumen 19. Oxine however, has been discontinued as a commercially available radiolabelling kit.

In this study, two novel exosome radiolabelling approaches using ^111^In^3+^ as the radioisotope were explored. One method involves radioisotope entrapment approach similar to oxine, but using tropolone, a safe and cheap alternative to oxine which has been associated with solubility and toxicity issues 43-46 as the ionophore for ^111^In^3+^ shuttling into the exosomal lumen. The second method involved covalently attaching DTPA-anhydride, a bifunctional chelator on the exosome surface that confers them the ability to bind ^111^In^3+^ stably. Although both of these approaches have been used to radiolabel cells, the properties of exosomes labelled by these means have not previously been evaluated. Therefore, the radiolabelling efficiency and stability of both approaches were assessed, and the approach with the most favourable outcome was used to study the biodistribution of melanoma-derived exosomes in both immunocompetent and immunodeficient melanoma bearing mice, to investigate the effect of the mouse immune system on exosome biodistribution.

## Materials and Methods

### Materials

Sterile Newborn Calf Serum Heat Inactivated was purchased from First Link (UK). Millex-GP Syringe Filter Units 0.22 µm were purchased from Merck Millipore (UK). Copper 300-mesh grid was purchased from Elektron Technologies (UK). Sodium chloride and glycine were purchased from VWR Chemicals (UK). CELLine AD1000 bioreactor flasks was purchased from Wheaton (UK). Indium-111 chloride was purchased form Mallinckrodt (NL). Sepharose® CL-2B was purchased from GE Healthcare Life Sciences (UK). Sucrose, chloroform, magnesium sulphate and acetic acid were purchased from Fisher Scientific (UK). Ammonium acetate was purchased from Santa Cruz Biotechnology (UK). Thin layer chromatography (TLC) papers were purchased from Agilent Technologies UK Ltd (UK). Isoflurane (IsoFlo®) for anaesthesia was purchased from Abbott Laboratories (UK). PBS pH 7.4 10X, Advanced RPMI, penicillin/Streptomycin, GlutaMax™ 100X, Trypsin- EDTA 0.05%, aldehyde/sulfate latex beads 4% w/v 4 µm and Micro BCA™ kit were purchased from Thermo Fisher Scientific (UK). Deuterium oxide, tropolone, diethylenetriaminepentaacetic dianhydride (DTPA-anhydride), Trypan blue 0.4%, D-(+)-Glucose 10%, Laminin, DMEM Nutrient Mixture F-12 Ham, BSA and HEPES buffer were purchased from Sigma- Aldrich (UK). Anti-CD81 and anti-CD9 polyclonal primary antibodies were purchased from Bioss antibodies (USA). Goat anti-rabbit secondary antibody Cy5-conjugated was purchased from Abcam (UK).

### Cell culture conditions

The murine melanoma B16F10 cells were cultured in Advanced RPMI 1640 medium supplemented with either 10% normal or exosome-depleted FBS, 1% penicillin/streptomycin and 1% GlutaMax™ in CELLine AD1000 bioreactor flasks. Exosome- depleted FBS was prepared by subjecting FBS to ultracentrifugation at 100,000 g for 18 h at 4°C. The FBS supernatant post-centrifugation was collected and sterile-filtered using 0.22 µm filters for use in cell culture. Cells from 4 x T75 flasks (80% confluent) in 15 ml medium supplemented with 10% exosome- depleted FBS were seeded into the cell compartment of 1 bioreactor flask. The medium reservoir compartment of the flask was filled with 500 ml of the medium supplemented with 10% normal FBS. Culture supernatant or conditioned medium (CM) was harvested from the cell compartment of the flask on a weekly basis and replaced with 15 ml of fresh medium supplemented with 10% exosome-depleted FBS. Collected CM was stored at 4°C until used for exosome isolation.

### Exosome isolation

B16F10 CM was pre-cleared of dead cells and cellular debris by several rounds of differential centrifugation: 500 g for 5 minutes at 4°C (twice), then at 2000 g for 15 minutes; followed by filtration through 0.22 µm filter. Pre-cleared CM (22.5 ml) was added to polycarbonate ultracentrifuge tubes (355631, Beckman Coulter). Sucrose solution (25% w/w in D_2_O, 3 ml) was then carefully added to the bottom of the CM using glass pipettes. The ultracentrifuge tubes were placed in a swing-out rotor (SW45 Ti, Beckman Coulter) and subjected to ultracentrifugation at 100,000 g for 90 min at 4°C (Optima™ XPN-80, Beckman Coulter). Post-centrifugation, the sucrose solution (2 ml) was withdrawn and added to 20 ml filtered PBS in polycarbonate ultracentrifuge bottles (355618), Beckman Coulter), and subjected to another round of ultracentrifugation in a fixed-angle rotor (Type 70 Ti, Beckman Coulter) at 100,000 g for 90 min at 4°C. The pellet obtained was resuspended in 400 µl filtered PBS.

### Nanoparticle tracking analysis (NTA) and protein measurements

Exosome hydrodynamic size and number were measured by nanoparticle tracking analysis (NTA) using NanoSight LM10 (Malvern Instruments, UK). The exosome sample was first diluted in filtered PBS to obtain 20-80 particles in the viewing frame. The modal size and particle count were measured in triplicates, with 30 s as the duration for each recording, and analysed using the NanoSight NTA 3.2 software (Malvern Instruments, UK). The results were expressed as mean ± standard deviation (SD). Protein measurements were measured using Micro BCA™ kit.

### Flow cytometry

Exosomes were coupled to latex microbeads using a protocol adapted from Théry et al. 47 prior to the detection of exosomal surface markers with flow cytometry. Briefly, 40 µl of exosomes were incubated with 10 µl aldehyde/sulphate latex beads for 15 min at room temperature (RT) before 5 µl of 100 µM BSA solution was added to the exosome-bead mixture (10 μM final concentration). This was followed by incubation in 1 ml glycine (100 mM in PBS) for 30 min at RT, after which it was centrifuged for 5 min at 580 g and washed twice with 1 ml of 3% exosome-depleted FBS (made in PBS, henceforth referred to as 3% FBS/PBS). After the second wash, the pellet was resuspended in 3% FBS/PBS and stained with CD81 and CD9 antibodies respectively (rabbit anti-mouse) followed by the Cy5-conjugated secondary antibody (45 min each at 4°C). The bead/ exosome complexes were washed once with 1 ml 3% FBS/PBS after incubation with each antibody, and the pellet resuspended in an appropriate volume of 3% FBS/PBS. The exosome-bead complex was run on FACSCalibur using FL4 channel for detection of Cy5 signals, and the results were analysed using CellQuest Pro software (BD Biosciences, US). A control sample consisting of beads only was prepared and subjected to the same treatment as the above but without staining.

### Transmission and scanning electron microscopy

For transmission electron microscopy (TEM), a sample of exosomes was fixed in formaldehyde/ glutaraldehyde (2.5% each in 0.1 M sodium cacodylate buffer, pH 7.4) for 15 min. The sample was then placed on 300-mesh carbon-coated copper grids and left to air-dry. Negative staining was achieved using filtered aqueous uranyl acetate (25% in methanol) for 4 min followed by two 50% methanol/H_2_O washes and left to air-dry. The grids were imaged using Philips CM 12 (FEI Electron Optics, NL) equipped with Tungsten filament and a Veleta - 2k x 2k side-mounted TEM CCD camera (Olympus, Japan) with the following settings: accelerating voltage - 80 kV; spot size - 2; objective aperture - 150 µm.

For scanning electron microscopy (SEM), a sample of exosomes was fixed in 5% glutaraldehyde for 2 h, which was then added on the surface of APTES pre-treated silicon wafer and left for 1 h. This was followed by washing with PBS three times and dehydrated in a series of increasing ethanol concentrations (20, 50, 70, 90, 95, 100%). The samples were transferred for critical drying (Samdri, Tousimis), and sputter coated with gold before scanning. SEM was performed on FEI Inspect-F (Philips, Eindhoven, NL) operated at 20 kV.

### Intraluminal radiolabelling of exosomes ([^111^In]-Exo_B16_)

Tropolone was dissolved in 200 mM HEPES buffered saline (HBS) pH 7-7.5 to make 1 mg/ml stock solution. 70-100 MBq ^111^InCl_3_ was added to 2 µg (2 μl) tropolone from the stock solution allowing for the [^111^In]Tropolone complex to form. The [^111^In]Trop mixture was added to exosomes (1 x 10^11^ particles/ mouse) diluted with PBS to achieve a final tropolone concentration of 5 µg/ml and incubated for 20 min at 37°C. Radiolabelled exosomes ([^111^In]-Exo_B16_) were purified from free [^111^In]Trop complex by gel filtration using Sepharose® CL-2B as the resolving matrix, self-packed according to the dimensions of the commercially available NAP-5™ columns, and optimised such that exosomes will elute in the first 2 x 500 µl fractions (F1 and F2). Radiolabelling efficiency was calculated as follows:


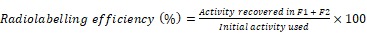


### Membrane radiolabelling of exosomes ([^111^In]DTPA-Exo_B16_)

DTPA-anhydride was added to dry chloroform (prepared by adding magnesium sulphate powder to chloroform and stirring the mixture vigorously for 2 min, and then filtered to remove the powder) to form a suspension at a concentration of 1 µg/µl, with brief sonication to break visible clumps. The amount required for the reaction with exosomes was added into a microtube and passed under a nitrogen stream to evaporate the chloroform thus forming a thin film of DTPA-anhydride on the lining of the microtube. Exosomes (1 x 10^11^ particles/mouse in 100 µl) were added to the DTPA-anhydride film at a molar ratio of 1:400 (lysine on exosome:anhydride - it was assumed that 1 exosome is equivalent to 1 BSA molecule i.e. containing 59 lysine residues) and incubated at 37°C for 30 min. Excess unreacted DTPA-anhydride was purified using Sepharose® CL-2B columns as described above, but using non-buffered saline as the elution buffer (and therefore the buffer to pre-equilibrate the column). 15-50 MBq of ^111^InCl_3_ was added to 0.2 M ammonium acetate buffer (pH 5.5) to achieve a final volume of 500 µl. This was then added to an equal volume of DTPA-Exo_B16_ to achieve a final concentration of 0.1 M ammonium acetate buffer (pH 5.5). The mixture was incubated for 5 min at RT. Radiolabelled exosomes ([^111^In]DTPA-Exo_B16_) were purified from excess ^111^InCl_3_ using Sepharose® CL-2B columns, this time using PBS as the elution buffer. The radiolabelling efficiency was then determined as described above.

### Radiochemical stability assessment

#### Intraluminal-labelled exosomes

[^111^In]-Exo_B16_ was incubated in 50% FBS or PBS (1:1, v/v) for 24 h at 37°C. Samples post-incubation were passed through Sepharose® CL-2B columns and the first 2 x 500 µl fractions (F1 and F2) were collected as described earlier. Radiochemical stability of [^111^In]-Exo_B16_ was calculated as follows:


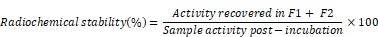


#### Membrane-labelled exosomes

[^111^In]DTPA-Exo_B16_ was incubated in 50% FBS or PBS as described above, and samples post-incubation were then spotted on thin layer chromatography (TLC) paper strips impregnated with silica gel. The strips were eluted with 0.1 M ammonium acetate containing 0.25 mM EDTA (pH 5.5) and analysed on a phosphor imager (Cyclone® Packard, Australia). The percentage of ^111^In still attached to exosomes (immobile spot at the application point) was considered as the radiochemically stable [^111^In]DTPA-Exo_B16_.

### Animal models

All animal experiments were performed in compliance with the UK Home Office Animals (Scientific Procedures) Act 1986. Female C57Bl/6 mice and male NOD SCID gamma (NSG) mice (~20 g, 6-8 weeks old) were obtained from Charles River (UK). Subcutaneous (SC) tumours were established by inoculating B16F10 cells (1 x 10^6^ cells in 100 µl PBS) subcutaneously into the left and right rear flanks of the mice. The mice were monitored closely post- inoculation and were used for studies when the tumours reached ~200-300 mm^3^.

### Whole body SPECT/CT imaging of radiolabelled exosomes

For intraluminal-labelled exosomes, C57Bl/6 mice (n=3 per treatment) was injected intravenously *via* the tail vein with 1 x 10^11^ [^111^In]-Exo_B16_ (5-10 MBq) or the equivalent amount of radioactivity of free [^111^In]Trop. For membrane-labelled exosomes, C57Bl/ 6 mice (n=3 per treatment) was injected with 1 x 10^11^ [^111^In]DTPA-Exo_B16_ (5 - 10 MBq) or the equivalent amount of radioactivity of free [^111^In]DTPA. Mice were imaged under anaesthesia (2% isoflurane in air) in prone position on a heating pad at 37°C using a nanoSPECT/CT four-head scanner (Bioscan, USA). SPECT images were obtained at 0-30 min, 4 h and 24 h post-injection using 1.4 mm pinhole collimators (24 projections, 60 s per projection; 30 min scan) and CT images were obtained at the end of each SPECT acquisition using an X-ray source setting of 45 kVp. All data were reconstructed with proprietary Bioscan software and SPECT and CT acquisitions were fused using PMOD® software (Mediso). Mice were culled and disposed of after the 24 h imaging.

### *Ex vivo* gamma counting of excised organs/tissue

Similar to SPECT/CT imaging, mice were injected intravenously with 1 x 10^11^ [^111^In]-Exo_B16_ (0.5 - 1 MBq) or free [^111^In]Trop of equivalent radioactivity; and 1 x 10^11^ [^111^In]DTPA-Exo_B16_ (0.5 - 1 MBq) or free [^111^In]DTPA of equivalent radioactivity. Blood samples (5 µl from the tail vein) were taken at various time points (2, 5, 10, 30, 60, 240 and 1440 min) to analyse the exosome circulation profile. Urine and faeces were collected by housing the mice in metabolic cages for 24 h to analyse the excretion profile. After 1, 4 and 24 h, mice were sacrificed (n=3 per time point) and perfused with heparinised saline (1000U/l, 25 ml per animal). Major organs (brain, lungs, liver, spleen, kidneys, heart, and stomach), muscle, skin, bone (femur), carcass and tumours were collected, weighed and placed in scintillation vials. Samples were counted in a gamma counter (LKB Wallac 1282 Compugamma, PerkinElmer, UK) together with radioactive dose standards. Radioactivity readings (counts per minute - CPM) were expressed as percentage of injected dose per organ (%ID/organ) or percentage of injected dose per gram of tissue (%ID/gT). Data were expressed as the mean ± SD of sample triplicates.

### Statistical analyses

For all experiments, data were presented as mean ± SD, where n denotes the number of repeats. Statistical significance of the data was assessed using Student's t-test and is designated with asterisk(s) (p* < 0.05, p**< 0.01, and p***< 0.001).

## Results

### Exosome isolation and physicochemical characterisation

Cancer cell lines are known to be good exosome producers 3-6 and hence they were selected as exosome sources in this study. Exosomes were isolated from B16F10 cells (murine melanoma) cultured in a bioreactor flask (CELLine AD1000) which can help increase the yield of exosomes 48. The culture supernatant, hereby referred to as conditioned medium (CM) was harvested on a weekly basis. CM initially underwent pre-clearing to remove dead cells and cellular debris by a series of differential centrifugation and ultrafiltration. Pre-cleared CM was then subjected to ultracentrifugation onto a sucrose cushion to separate exosome from proteins by density in the CM, followed by a washing step to remove the sucrose and residual contaminating proteins. The resulting exosome pellet was resuspended in a small volume of PBS (400 µl) to make a concentrated exosome stock.

The physicochemical characterisation of exosomes isolated from B16F10 (Exo_B16_) cells are summarised in **Table 1**. The size measured using NTA was 132.3 ± 5.6 nm, which compares to other exosome studies. Particle concentration quantification (also using NTA) showed that B16F10 cells are a prolific exosome producer, yielding 2.04 x 10^13^ ± 3.9 x 10^12^ p/ml from 72 ml of CM (obtained from 6 rounds of CM collection from a single bioreactor flask). A measure of purity of the Exo_B16_ from contaminating proteins was also carried out by means of calculating the particle to protein (P:P) ratio of the isolated exosome stock. The P:P ratio was found to be 4.52 x 10^10^ ± 1.26 x 10^10^ p/µg protein, which falls in the proposed range of high purity level 48.

### Biochemical and morphological analysis of Exo_B16_

**Figure 1:**
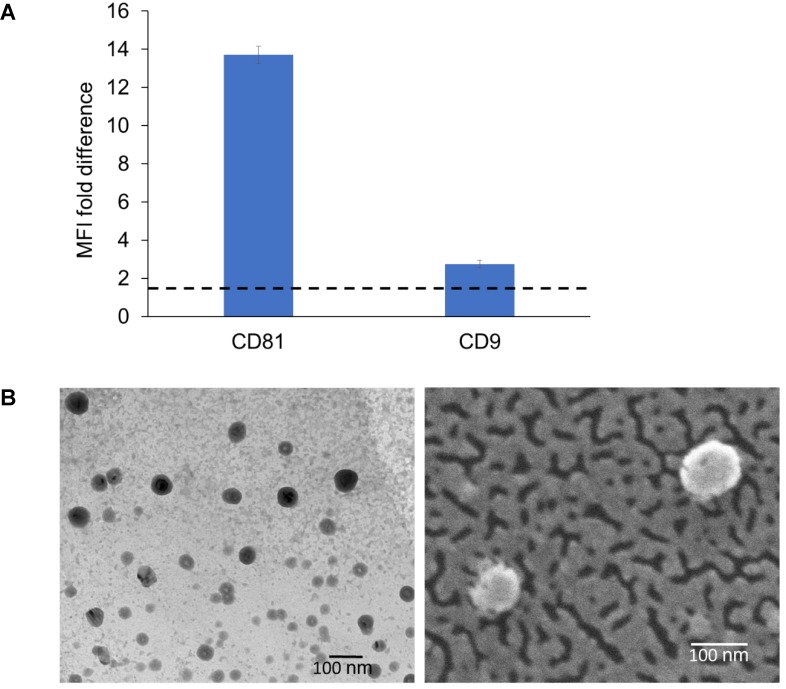
** Biochemical and morphology analysis of B16F10 exosomes. (A)** Detection of exosomal markers CD81 and CD9 using flow cytometry on exosomes isolated from B16F10 cells. Exosomes were coupled to aldehyde/sulphate latex beads prior to detection. Exo-beads complex were subsequently stained using a 2-step labelling (anti-CD81 or anti-CD9 1° ab/Cy5-conjugated 2° ab). Degree of expression of the markers are expressed as the fold difference in median fluorescence intensity (MFI) values from that of the control (exo-beads complex stained with Cy5-conjugated 2° ab). Values are expressed as mean ± SD, where n=3. **(B)** Transmission electron microscopy (TEM) and scanning electron microscopy (SEM) images of exosomes isolated from B16F10 cells.

Detection of exosomal markers was achieved using flow cytometry as previously described 49. The isolated Exo_B16_ expressed CD81 and CD9, which is a common property of exosomal vesicles (**Fig. 1A** and** Fig. S1)**. Morphological analysis of Exo_B16_ was also undertaken using both transmission electron microscopy (TEM) and scanning electron microscopy (SEM), which can also validate the size measurement obtained from NTA. Both TEM and SEM images of Exo_B16_ demonstrated that the exosomes were spherical structures slightly above 100 nm in size **(Fig. 1B)**.

### Intraluminal radiolabelling of Exo_B16_

#### Radiolabelling efficiency and stability

**Scheme 1:**
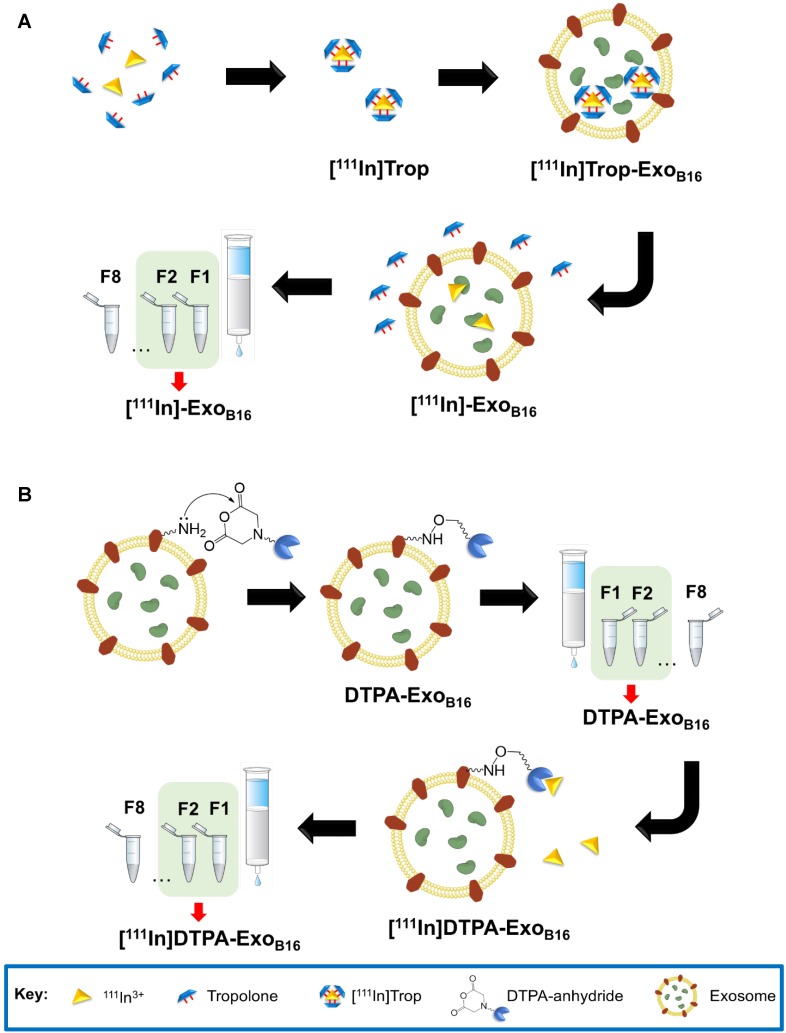
** (A)** Intraluminal and **(B)** membrane radiolabelling protocols of B16F10 exosomes

The intraluminal radiolabelling approach is achieved *via* the ability of the small hydrophobic molecule called tropolone to chelate radionuclides and form a complex that allows the radionuclide to diffuse across the exosomal membrane and into the exosomal lumen, similar to its predecessor oxine 50. This method, particularly utilising the ^111^Indium- tropolone complex ([^111^In]Trop) has been widely used to radiolabel cellular components of blood such as platelets 43, 44, lymphocytes 45, 51 and granulocytes 52 for *in vivo* imaging. Other cell types such as mesenchymal stem cells 53, 54 and endometrial cells 55 have also been successfully radiolabelled using [^111^In] Trop. More recently, [^111^In]Trop was used to label polymeric micelles 56. The orientation of exosomal transmembrane proteins is the same as their parent cells 15 and therefore provides the opportunity for them to be radiolabelled using the same principle. The mechanism by which this intraluminal radiolabelling is achieved is summarised in **Scheme 1A**. Tropolone is firstly mixed with ^111^Indium (as ^111^InCl_3_) to allow for the formation of the of the [^111^In]tropolone complex. The chemical structure of tropolone and [^111^In]Trop complex is illustrated in **Scheme S1A** and **S1B** (supplementary information). Upon incubation with exosomes, [^111^In] Trop gets translocated into the exosomal lumen, forming the intermediate [^111^In]Trop-Exo_B16_. Upon entry, ^111^In^3+^ exchanges to bind with cytoplasmic biomolecules of at least 3.6 kDa 50. As the interaction between ^111^In^3+^ and tropolone is not particularly strong, ^111^In^3+^ from the [^111^In]Trop will then exchange with proteins and nucleic acids within the exosomal lumen 50. Free tropolone molecules leave the exosomal lumen and the ^111^In^3+^ is now entrapped within the lumen, thereby resulting in radiolabelled exosomes ([^111^In]-Exo_B16_).

**Figure 2:**
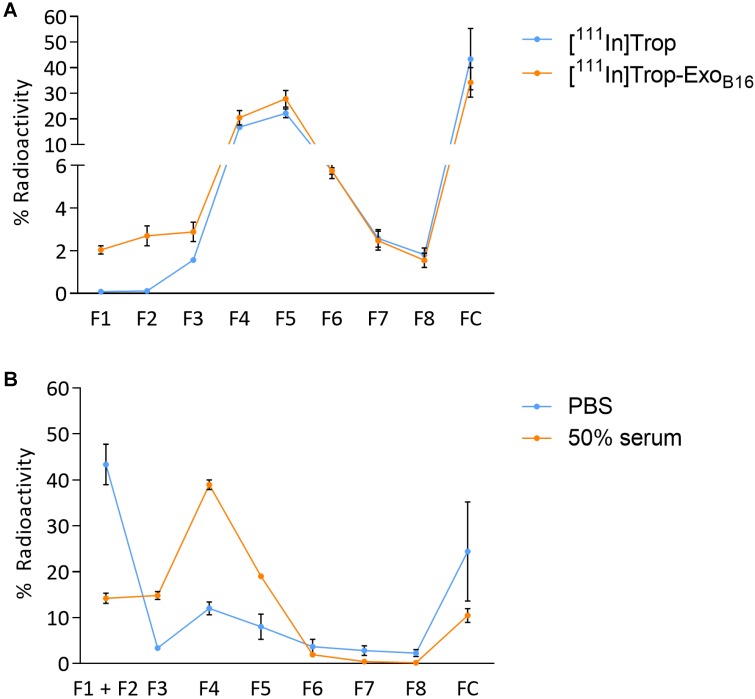
** Radiolabelling efficiency and radiochemical stability of intraluminal-labelled B16F10 exosomes. (A)** Radiolabelled exosomes ([^111^In]-Exo_B16_) were purified from excess [^111^In]Trop complex by gel filtration (Sepharose® CL-2B). Eight 500 µl fractions were collected and the radioactivity for each fraction and the column itself post-purification was measured by gamma counting, and are expressed as the percentage of activity relative to the initial activity added to the column. Radiolabelling efficiency was calculated as the sum of % radioactivity recovered from F1 and F2. **(B)** Radiolabelled exosomes were incubated in either PBS or 50% serum for 24 h at 37°C, and then passed through the same column as **(A)**. Eight 500 µl fractions were collected and the radioactivity for each fraction and the column itself (FC) post-purification was measured using gamma counter, and are expressed as the percentage of activity relative to the activity of the sample added to the column. Radiochemical stability was calculated as the sum of % radioactivity recovered from F1 and F2. Values are expressed as mean ± SD, where n=3. Statistical analysis was done on F1 and F2 (p* < 0.05, p*** <0.001).

Purification of excess [^111^In]Trop from [^111^In]- Exo_B16_ was carried out *via* gel filtration using Sepharose® CL-2B as the resin. Free [^111^In]Trop was used as a control and the elution profiles of both [^111^In]-Exo_B16_ and [^111^In]Trop were analysed. Exosomes were found to elute in F1 and F2 (**Fig. S2A and S2B)** while [^111^In]Trop mainly eluted in F4 and F5 (**Fig. 2A)**. Radiolabelling efficiency for [^111^In]-Exo_B16_ was 4.73 ± 0.39% compared to only 0.20 ± 0.04% (p<0.05) for [^111^In]Trop collected in F1+F2 **(Fig. 2A)**.

[^111^In]-Exo_B16_ were incubated in either PBS or 50% serum at 37°C for 24 h to assess the radiochemical stability of the labelling. The typical method of assessing the labelling stability is using thin layer chromatography (TLC), by measuring the % activity that did not migrate with the mobile phase and remains at the application point after 24 h incubation, corresponding to radiolabelled macromolecules 57-59. It was not possible to apply this method to assess the intraluminal labelling stability of exosomes as [^111^In]Trop will not migrate using 0.1 M ammonium acetate with 0.25 mM EDTA, pH 5.5 mobile phase, rendering it impossible discern whether the % activity remaining at the application point is originating from that of radiolabelled exosomes or free [^111^In]Trop. An alternative approach was used by passing sample post-incubation through Sepharose® CL-2B column used previously to determine radiolabelling efficiency. The stability of intraluminal-labelled [^111^In]-Exo_B16_ was found to be 43.35 ± 10.12% and 14.21 ± 2.76% in PBS and 50% serum, respectively, at 24 h post incubation **(Fig. 2B)**.

Biodistribution of intraluminal-labelled [^111^In]- Exo_B16_ was assessed qualitatively and quantitatively using whole body SPECT/CT imaging and *ex vivo* gamma counting, respectively. Significant difference was observed in the organ accumulation profile of [^111^In]-Exo_B16_ as compared to that of free [^111^In]Trop, which further supports the successful radiolabelling of the exosomes (**Fig. S3, S4 and S5**). However, due to the low stability of the intraluminal-labelled [^111^In]-Exo_B16,_ the reliability and accuracy of the organ accumulation values, especially that of the tumours were deemed improbable.

### Membrane radiolabelling of Exo_B16_

#### Radiolabelling efficiency and stability

Membrane radiolabelling was achieved by covalently attaching the bifunctional chelator DTPA- anhydride to the exosome surface in an amine- dependent reaction. Exosome membranes contain various transmembrane proteins which are likely to have free amines (from lysine residues) on the extraluminal domain. The schematics of the reaction are summarised in **Scheme 1B**. The free amines act as nucleophiles that attack anhydrides on the DTPA, resulting in exosomes with covalently attached DTPA on their surface *via* amide bonds (DTPA-Exo_B16_). Incubating DTPA-Exo_B16_ with ^111^Indium (as ^111^InCl_3_) will then allow ^111^In^3+^ to be chelated by DTPA on the exosomes, thereby radiolabelling the exosomes ([^111^In]DTPA-Exo_B16_). The chemical structure of DTPA-anhydride and its reaction with exosomal amine is illustrated in **Scheme S1C** and **S1D** respectively (supplementary information).

**Figure 3:**
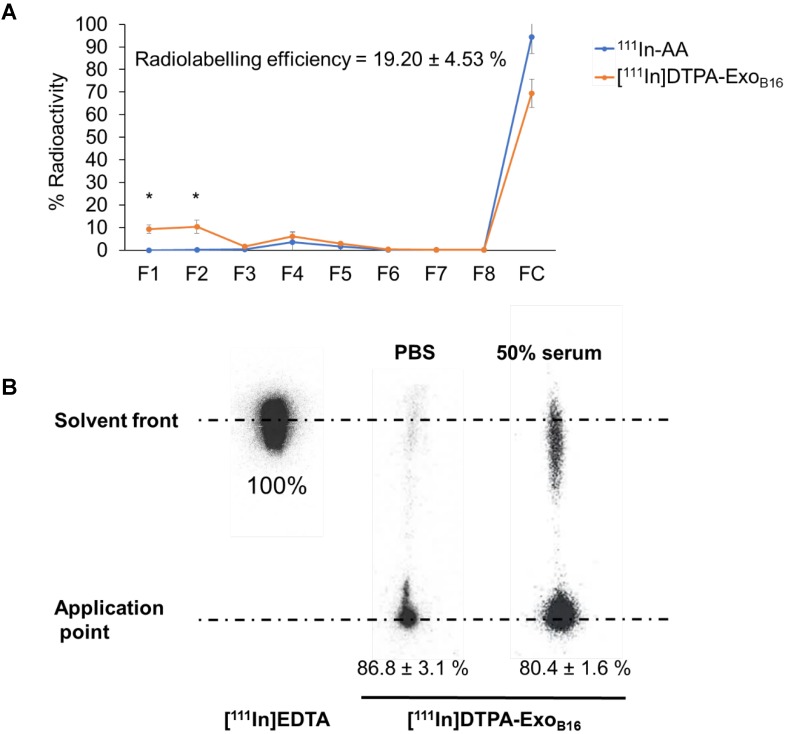
** Radiolabelling efficiency and radiochemical stability of membrane-labelled B16F10 exosomes. (A)** B16F10 exosomes with covalently attached DTPA (DTPA-Exo_B16_) were purified from excess ^111^InCl_3_ by gel filtration (Sepharose® CL-2B). Free ^111^In in ammonium acetate buffer (^111^In-AA) was also eluted through the column as control. Eight 500 µl fractions were collected and the radioactivity for each fraction and the column itself post-purification was measured by gamma counting, and are expressed as the percentage of activity relative to the initial activity added to the column. Radiolabelling efficiency was calculated as the sum of % radioactivity recovered from F1 and F2. Values are expressed as mean ± SD, where n=3. Statistical analysis was done on F1 and F2 (p** < 0.01, p*** <0.001). **(B)** Radiolabelled exosomes ([^111^In]DTPA-Exo_B16_) were incubated in either PBS or 50% serum for 24 h at 37°C, and then spotted on a TLC paper. The paper was then run on 0.1 M ammonium acetate with 0.25mM EDTA (pH 5.5) as the mobile phase and imaged using a phosphorimager. Radiochemical stability was calculated as % radioactivity remaining at the application point. Values are expressed as mean ± SD, where n=3.

Radiolabelling efficiency of membrane-labelled exosomes was assessed in a similar manner to that of intraluminal labelling, where free ^111^In in 0.2M ammonium acetate buffer pH 5.5 (^111^In-AA) and [^111^In]DTPA-Exo_B16_ were eluted through Sepharose® CL-2B columns and their respective elution profiles analysed. Radiolabelling efficiency was determined as the % radioactivity recovered in F1+F2. Percentage (%) activity recovered in F1+F2 for [^111^In]DTPA-Exo_B16_ was significantly higher than that of ^111^In-AA (19.2 ± 4.53% and 0.02 ± 0.001% respectively), thereby confirming that the activity recovered in F1+F2 were from [^111^In]DTPA-Exo_B16_ **(Fig. 3A)**. The radiolabelling efficiency of [^111^In]DTPA-Exo_B16_ considered to be 19.2 ± 4.53 % was significantly higher than that obtained by intraluminal-labelling method **(Fig. 2A)**.

Radiochemical stability of [^111^In]DTPA-Exo_B16_ in PBS and 50% serum after 24 h at 37ºC was assessed using TLC as explained above, by measuring the % activity that did not migrate with the mobile phase (0.1 M ammonium acetate with 0.25 mM EDTA, pH 5.5) and remained at the application point, corresponding to radiolabelled exosomes. Free In^3+^ was also run on the TLC paper as a control, where they all migrate to the solvent front as they were chelated by EDTA present in the mobile phase **(Fig. 3B)**. The stability of [^111^In]DTPA-Exo_B16_ in PBS and 50% serum was ~87 % and ~80% respectively, both higher than that of intraluminal-labelled exosomes **(Fig. 2B)**.

#### Whole body SPECT/CT live imaging

**Figure 4:**
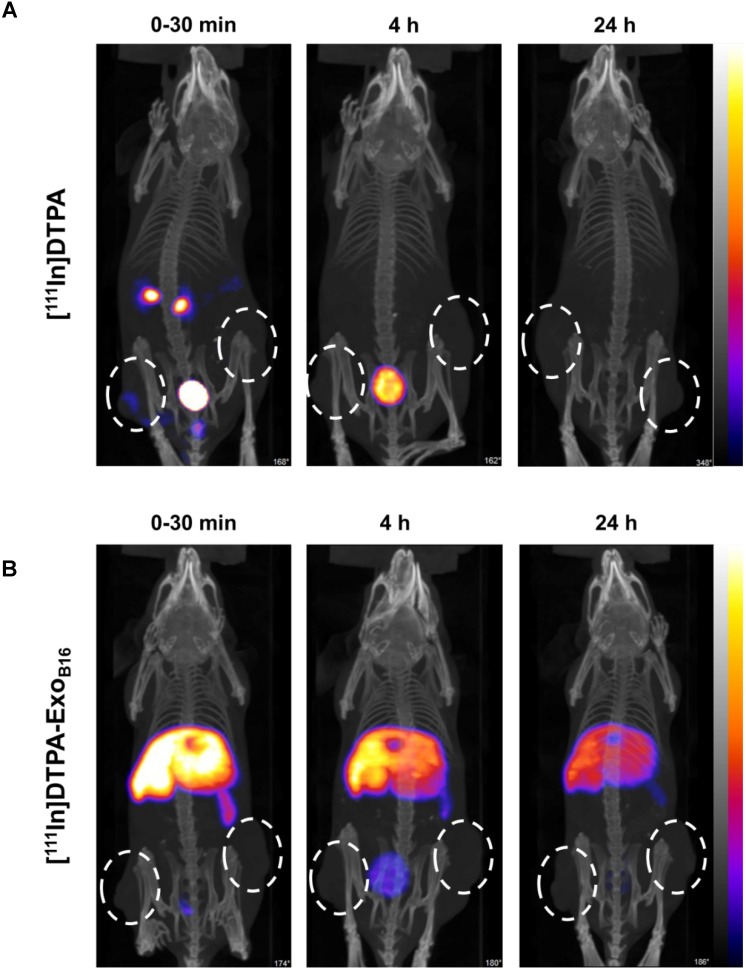
** Whole body SPECT/CT imaging of membrane-labelled B16F10 exosomes in melanoma-bearing C57Bl/6 mice. (A)** Animal was injected intravenously with free [^111^In]DTPA complex as control. **(B)** Animal was injected with [^111^In]DTPA-Exo_B16_. Imaging was done immediately, 4, and 24 h post-injection. White circles indicate the position of tumours.

Free [^111^In]DTPA and [^111^In]DTPA-Exo_B16_ were injected intravenously into C57Bl/6 mice bearing subcutaneous B16F10 tumours (1x10^11^ particles per animal for [^111^In]DTPA-Exo_B16_) for whole body SPECT/CT imaging, of which the former acts as a control to ensure the signals detected *in vivo* were coming from [^111^In]DTPA- Exo_B16_ and not from free circulating [^111^In]DTPA that was cleaved from the exosome surface. Imaging was undertaken immediately, 4 h and 24 h post- injection. Imaging results showed a significant difference between the biodistribution profile of [^111^In]DTPA and [^111^In]DTPA- Exo_B16_ **(Fig. 4)**. At 0-30 min post-injection, high amounts of [^111^In]DTPA can be seen in kidneys and bladder indicating high urinary excretion, but at 4 h the signals can only be seen in the bladder, which then becomes too low to be detected at 24 h. In contrast, [^111^In]DTPA-Exo_B16_ showed very high signals in the liver and spleen at 0-30 min post-injection, which remained up to 24 h. Some signals were detected in the bladder at the earlier timepoints, and tumour accumulation was not observed. The clear distinction between the *in vivo* imaging results of [^111^In]DTPA and [^111^In]DTPA-Exo_B16_ corroborated the finding that the % activity recovered in F1+F2 from **Fig. 3A** originated from labelled exosomes and confirms successful membrane radiolabelling of exosomes.

#### Quantitative organ biodistribution by gamma counting

**Figure 5:**
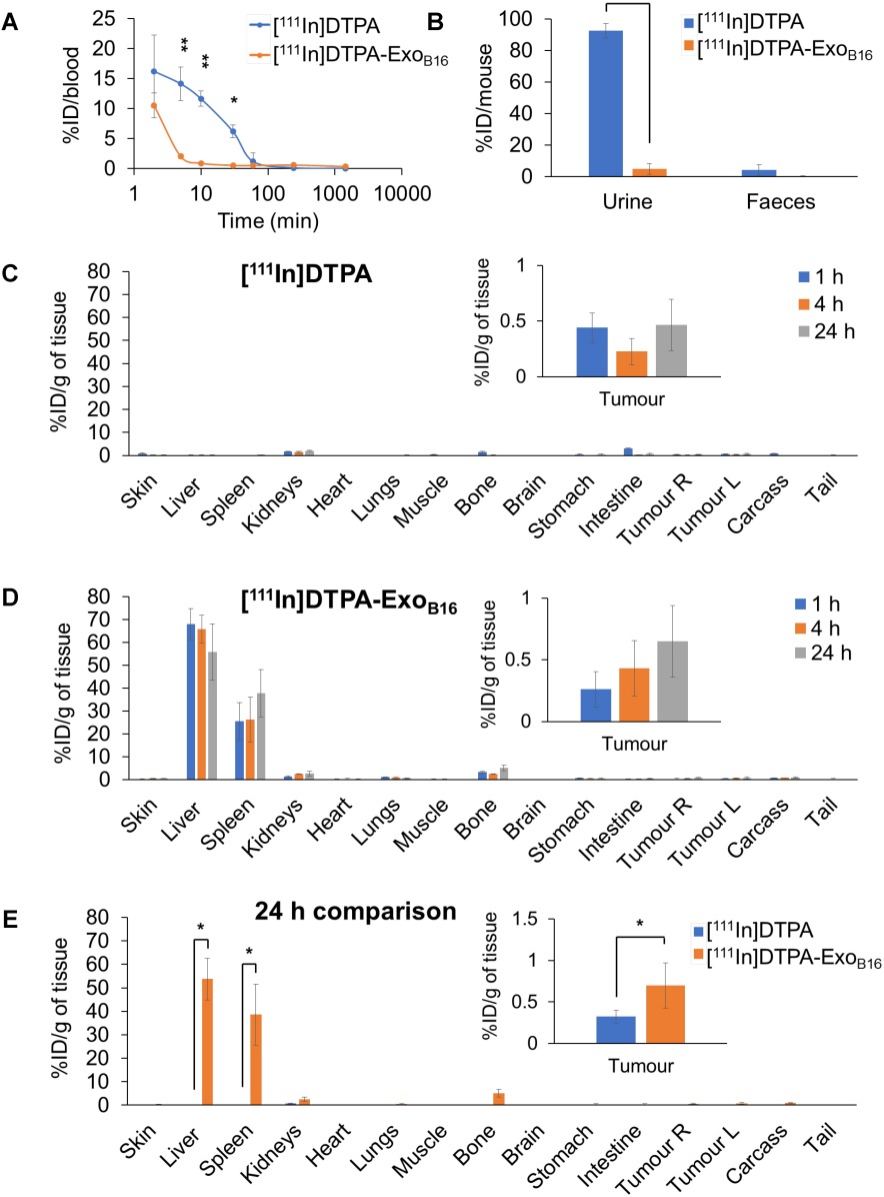
** Blood circulation, excretion and organ biodistribution profiles of membrane-labelled B16F10 exosomes in melanoma-bearing C57Bl/6 mice.** The [^111^In]DTPA group was injected with 0.02M DTPA complexed with 0.5-1MBq of ^111^InCl_3_ while the [^111^In]DTPA- Exo_B16_ group was injected with 1x10^11^ [^111^In]DTPA-Exo_B16_ (0.5-1MBq). **(A)** Blood circulation profile of [^111^In]DTPA and [^111^In]DTPA-Exo_B16_. 5 µl blood were taken *via* tail bleeding at 2 min, 5 min, 10 min, 30 min, 1 h, 4 h and 24 h following intravenous injection of each compound. **(B)** Excretion profile of [^111^In]DTPA and [^111^In]DTPA-Exo_B16_ where urine and faeces were collected from the animals 24 h post-injection. **(C)** and **(D)** Organ biodistribution of [^111^In]DTPA and [^111^In]DTPA-Exo_B16_ respectively. Animals were culled at 1 h, 4 h and 24 h post-injection, perfused with saline and their organs were excised for analysis by gamma counting. Inset shows the zoomed-in tumour accumulation values for each group. **(E)** Comparison of organ biodistribution of [^111^In]DTPA and [^111^In]DTPA-Exo_B16_ 24 h post-injection, where inset shows zoomed-in tumour accumulation values for each group. Values are normalised to organ weight and expressed as mean ± SD, where n=3 for each group. For **(C), (D)** and **(E),** statistical analyses were done on liver, spleen, kidneys and tumour (p*<0.05, p** < 0.01, p*** <0.001).

Quantitative biodistribution analysis of both [^111^In]DTPA and [^111^In]DTPA-Exo_B16_ at 1, 4 and 24 h was also carried out by gamma counting as per described above. Both [^111^In]DTPA and [^111^In]DTPA- Exo_B16_ were found to be cleared rapidly from the circulation with only ~16.2% injected dose (ID) and ~10.5% ID respectively remaining after only 2 min post-injection and reaching a very low level of 1% or slightly less in just 1 h **(Fig. 5A)**. Although both free [^111^In]DTPA and [^111^In]DTPA-Exo_B16_ showed rapid clearance from the circulation, they showed significantly different kinetics especially in the earlier timepoints (<60 min). As expected, a significantly higher amount of [^111^In]DTPA (~92.6% ID) was excreted in the urine as compared to that of [^111^In]DTPA-Exo_B16_ (~4.93% ID), but the amount excreted in faeces was similarly low for both compounds with a value of ~1-4 % ID **(Fig. 5B)**. Looking at organ biodistribution, there was minimal accumulation of [^111^In]DTPA with ~2.0 % ID per gram tissue (ID/gT) or lower across all organs including tumours, which recorded a value of about ~0.2 - 0.4 % ID/gT **(Fig. 5C)**. In contrast, [^111^In]DTPA-Exo_B16_ showed high accumulation in the liver with ~66.0% ID/gT at 1 and 4 h, which then decreased slightly to ~56.0 % ID/gT after 24 h, of which the difference is not significant **(Fig. 5D)**. This was followed by spleen, which showed an accumulation of ~26.0% ID/gT at 1 and 4 h, which increased slightly but not significantly to ~37.7% ID/gT. Kidneys showed an accumulation of ~2.5 % ID/gT of [^111^In]DTPA-Exo_B16_ after 24 h. Tumour accumulation of [^111^In]DTPA-Exo_B16_ was initially very low but showed a steady increase, reaching a value of ~0.7 % ID/gT after 24 h. When comparing organ biodistribution values at 24 h, a significant difference can be observed between that of [^111^In]DTPA and [^111^In]DTPA-Exo_B16_, where the latter showed a significantly higher liver, spleen and tumour accumulation as compared to that of the former **(Fig. 5E)**, which reflects successful and stable radiolabelling of Exo_B16_. The quantitative biodistribution results of both free [^111^In]DTPA and [^111^In]DTPA-Exo_B16_ expressed as %ID/organ are summarised in **Fig. S6** (supplementary information). In summary, results from membrane-labelled exosomes, especially the quantitative organ accumulation values, were deemed as more reliable due to its superior radiochemical stability (thus higher signal-to-noise ratio) and is selected as the approach for the subsequent part of the study.

### Comparative biodistribution of Exo_B16_ in immunocompetent and immunodeficient mice

**Figure 6:**
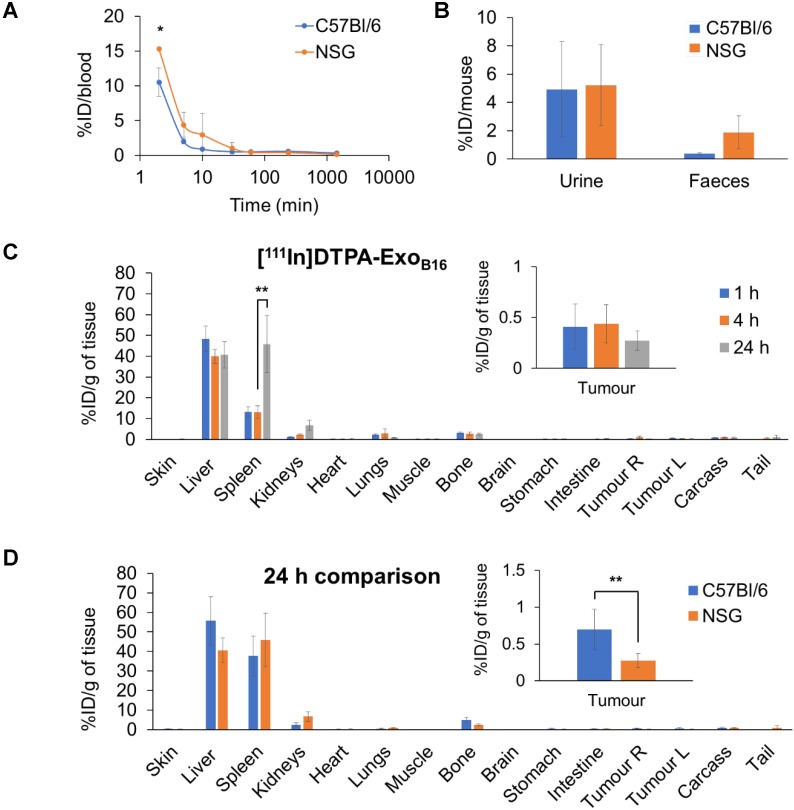
** Blood circulation, excretion and organ biodistribution profile of membrane-labelled B16F10 exosomes in melanoma-bearing NSG mice.** Animals were injected with 1x10^11^ [^111^In]DTPA-Exo_B16_ (0.5-1MBq). **(A)** Blood circulation profile of [^111^In]DTPA-Exo_B16 in_ NSG mice. Blood (5 µl) was taken *via* tail bleeding at 2 min, 5 min, 10 min, 30 min, 1 h, 4 h and 24 h following intravenous injection of exosomes. **(B)** Excretion profile of [^111^In]DTPA-Exo_B16_ in NSG mice where urine and faeces were collected from the animals 24 h post-injection. For **(A)** and **(B),** the values are plotted in comparison with that of C57BL/6 presented in **Fig. 5. (C)** Organ biodistribution of [^111^In]DTPA-Exo_B16_ in NSG mice. Animals were culled at 1 h, 4 h and 24 h post-injection, perfused with saline and their organs were excised for analysis by gamma counting. Inset shows the zoomed-in tumour accumulation values for each group. **(D)** Comparison of organ biodistribution of [^111^In]DTPA-Exo_B16_ in C57Bl/6 and NSG mice 24 h post-injection, where inset shows zoomed-in tumour accumulation values for each group. Values are normalised to organ weight and expressed as mean ± SD, where n=3 for each group. For **(C)** and **(D),** statistical analyses were done on liver, spleen, kidneys and tumour (p*<0.05, p** < 0.01, p*** <0.001).

Next, membrane-labelled exosomes were injected into NOD-*scid ILR2γ^null^* (NSG) mice to study the influence of the immune system on the *in vivo* biodistribution of exosomes. [^111^In]DTPA-Exo_B16_ (1x10^11^ particles per animal) were injected intravenously into NSG mice bearing subcutaneous B16F10 tumours and quantitative biodistribution analysis at 1, 4 and 24 h was carried out as the above *via* gamma counting. [^111^In]DTPA-Exo_B16_ showed a similar blood circulation profile in NSG mice when compared to that in C57Bl/6 mice, where [^111^In]DTPA-Exo_B16_ was cleared rapidly from the circulation with only ~15.3% ID remaining at 2 min post-injection, which decreased to ~0.1% ID after 24 h **(Fig. 6A).** [^111^In]DTPA-Exo_B16_ in NSG mice also showed similar amounts of excretion via the urine and faeces to that of C57BL/6 mice **(Fig. 6B)**. [^111^In]DTPA-Exo_B16_ in NSG mice also showed a similar pattern of organ accumulation to that in C57BL/6 mice across the time points, where the liver recorded the highest signals with ~48.3% ID/gT at 1 h, which then decreased slightly to ~40.0% ID/gT at 4 and 24 h **(Fig. 6C)**. Spleen recorded the second highest accumulation of ~13.0 % ID/gT at 1 and 4 h, which then increased significantly to ~45.8 % after 24 h. [^111^In]DTPA-Exo_B16_ accumulation in the kidneys showed a steady increase over the timepoints, reaching a value of ~6.8 % ID/gT after 24 h. The tumours showed an accumulation of around ~0.4 % ID/gT at 1 and 4 h, which then decreased slightly to ~0.3 % ID/gT after 24 h. Comparing biodistribution values at 24 h showed that [^111^In]DTPA-Exo_B16_ accumulation in C57BL/6 and NSG mice recorded similar values across the different organs, especially in the liver, spleen and kidneys **(Fig. 6D)**. [^111^In]DTPA-Exo_B16_ in NSG mice however showed a significantly lower tumour accumulation as compared to that in C57BL/6 mice. In summary, exosomes show a similar pattern of organ accumulation in both immunocompetent and immunodeficient mouse models with the exception of tumour, in which the accumulation in the latter was lower.

## Discussion

As described earlier, exosomes are very similar to cells in terms of being a phospholipid bilayer system, having the same membrane topology as their parent cells and inherently containing biomolecules such as proteins and RNAs as their cargo 15. Therefore, [^111^In]Trop was hypothesised to result in successful radiolabelling of exosomes. This approach did lead to successful radiolabelling of the exosomes as described earlier, but at a much lower efficiency compared to that observed in platelets of ~60-80% 43. Assuming platelet size to be 1 µm in diameter 60 and that of Exo_B16_ is ~130 nm **(Table 1)**, and that both are perfect spheres, the number of Exo_B16_ used in this study to determine the radiolabelling efficiency (3 x 10^11^ particles) accounts for a total surface area which is about double that of the platelets used in the above study (2.2 x 10^9^ platelets). However, the volume of an Exo_B16_ particle is ~45 times lower than that of a single platelet. Therefore, although [^111^In] Trop complexes were able to translocate efficiently into the exosomal lumen due to the large total surface area, their significantly lower volume suggests a much lower amount of biomolecules within the exosomal lumen for ^111^In^3+^ to exchange with as compared to that in the cytoplasm of platelets. ^111^In^3+^ translocated into the exosomal lumen probably largely still exists as [^111^In]Trop due to the lack of biomolecules for exchange and are well able to leave the exosomal lumen, forming an equilibrium in terms of its concentration within and outside the lumen, contributing to the low radiolabelling efficiency and stability. This is corroborated by the similar radioactivity detected in the tumours of mice injected with ^111^In-Exo_B16_ 24 h post-injection and that of the mice injected with free [^111^In]Trop, suggesting that the unexchanged ^111^In^3+^ in the form of [^111^In]Trop leaked out from the exosomal lumen into the circulation and gradually accumulated in the tumour **(Fig. S4C & S4D)**. Another possibility for the low serum stability observed with intraluminal-labelled exosomes is that the serum might be damaging the vesicles, thereby releasing the entrapped [^111^In]Trop. However, this possibility is ruled out as very good serum stability was observed with the membrane-labelled exosomes **(Fig. 3B)**.

The membrane or surface radiolabelling approach has been employed in synthetic nanocarriers such as polymeric nanocapsules and liposomes, whereby strong radioisotope chelators such as DTPA is incorporated as an integral component of their polymeric shell or membrane respectively during synthesis. This allows the nanocarriers to be radiolabelled when incubated with radioisotopes such as ^111^In^3+^, with their radiolabelling efficiency and stability reported to be between 61.9-100% and 78.2-91.3% respectively 57, 58, 61. This strategy of DTPA incorporation however is not possible on biomolecules such as exosomes, and so bifunctional chelators are used instead. Bifunctional chelators are molecules that consist of a strong chelating agent such as DTPA on one end, and a biologically-reactive functional group on the other end, usually amine- reactive groups such as NHS-ester or anhydride, or thiol-reactive groups such as maleimide 62. One such bifunctional chelator, cyclic DTPA- dianhydride (hereon referred to as DTPA-anhydride) was successfully conjugated to human serum albumin in a simple and rapid reaction, which allows subsequent radiolabelling with ^111^In^3+^ and its biodistribution analysed quantitatively 63. DTPA- anhydride has since been demonstrated to be successfully conjugated to other biomolecules such as fibrinogen 64 and antibodies 65-68 without losing their specificity and function, enabling quantitative analysis of their biodistribution. This same DTPA-anhydride, which is now commercially available, was adopted in this study where it was conjugated to Exo_B16_, and a 5-fold increase in both radiolabelling efficiency and stability was observed **(Fig. 3A & 3B)**.

Radiolabelling stability of [^111^In] DTPA-Exo_B16_ was similar to that reported in the studies above, and this was expected as DTPA is attached to exosomes *via* the same stable amide bond. The radiolabelling efficiency of [^111^In]DTPA-Exo_B16_ however is lower than that reported for the other nanocarriers, and this could be due to a lower number of DTPA molecules conjugated to Exo_B16_. In principle, this could be overcome by increasing the molar ratio of DTPA- anhydride in the reaction with the exosomes. However this would pose a problem due to rendering the reaction mixture more acidic due to the increasing amount of free DTPA forming from spontaneous hydrolysis in aqueous solution 68. Given that the pKa of the side-chain amine on a lysine residue is ~10.5, low pH conditions would easily increase the proportion of the protonated form of the free amines of the lysine residues on the exosomal surface, making them weaker nucleophiles and thereby reducing the efficiency of the reaction 68. This low pH could also adversely affect the function and integrity of other exosomal transmembrane proteins, which could affect the biodistribution as they have been reported to play a role in cell uptake 69-71. This is corroborated by studies that reported antibodies reacted with a high ratio of DTPA-anhydride had reduced antigen binding ability 65, 68. It is therefore important to determine the number of free amines on exosomal surface prior to the reaction with DTPA. This is challenging due to the heterogeneity of exosomes, even the ones isolated from the same source. In this study, the number of free amines i.e. lysine residues on exosomes was assumed to be similar to that of bovine serum albumin (BSA) and this was used as the basis for reaction with DTPA-anhydride. In our hands, reactions using 1:80, 1:200, 1:400 and 1:800 (Lys:anhydride) molar ratios showed increasing radiolabelling efficiency with increasing molar ratio up to 1:400 (data not shown) after which a decline was observed. The molar ratio 1:400 was therefore chosen for the DTPA-Exo_B16_ conjugation in this study.

Contaminating proteins from serum used in culture such as albumin and present in the Exo_B16_ sample 48, are likely to compete with the exosomes for the reaction with DTPA-anhydride 63, thus lowering Exo_B16_ labelling efficiency. Purifying exosome samples by gel filtration (e.g. Sepharose® CL-2B used in this study) or centrifugal filters (e.g. Nanosep®) prior to the labelling reaction can significantly reduce the amount of contaminating proteins in the sample, but this results in substantial loss of ~50% exosomes post-purification **(Fig. S7A and Fig. S7B)**. This can pose a serious challenge when working with a limited number of exosomes either obtained from cell cultures or liquid biopsies. Gel filtration using Sepharose® CL-2B resin as the resolving matrix was chosen in this study due to its superior contaminating protein removal performance and thus significantly better improvement in the P:P ratio of the exosome sample, without altering the size of the exosomes **(Fig. S7B, Fig. S7C and Fig. S7D)**. Hence, in case that DTPA-conjugated proteins were formed, they could be efficiently removed along with excess unreacted DTPA-anhydride post-reaction prior to incubation with ^111^In^3+^. The radiolabelling efficiency of [^111^In]DTPA-Exo_B16_ achieved in this study **(Fig. 3A)** was compliant to the “As Low As Reasonably Practicable” (ALARP) principle outlined by the UK's Health and Safety Executive (HSE) in terms of radioactivity required to perform SPECT/CT imaging and quantitative biodistribution studies.

As mentioned earlier, exosomal surface proteins play an important role in their interaction and subsequent uptake into cells, and that the disruption of these proteins can influence their tissue uptake/localisation *in vitro* and *in vivo* 69, 71, 72. DTPA-anhydride conjugation to exosomal surface proteins in the membrane labelling approach harbours such risk and could potentially influence the organ biodistribution of Exo_B16._ A dot blot analysis was carried out on the exosomes following a mock-radiolabelling protocol. It was found that surface proteins such as CD63 and CD9 are still present on the exosomes post-labelling, but showed a lower signal intensity upon detection **(Fig. S8)**. This is most probably due to the conjugated DTPA causing slight hindering of the antibody binding. Further studies have to be carried out to investigate whether this would influence exosome uptake and accumulation in tissues *in vivo*.

In this study, Exo_B16_ showed rapid clearance from the circulation, accumulating predominantly in the liver and spleen. This accumulation profile is consistent with a number of other exosome biodistribution studies involving optical and nuclear modalities, where usually kidneys were reported to show the 3^rd^ highest accumulation after the liver and spleen 19, 32-34, 73. Other types of nanocarriers bearing physicochemical resemblance to exosomes such as liposomes and polymeric nanocapsules were also reported to accumulate mostly in the liver and spleen 57, 58, 61, which further supports the findings of this study. Lung accumulation is more commonly observed for non-spherical carbon-based nanocarriers with high aspect ratio or surface area such as carbon nanotubes and graphene 59, 74, but a number of studies using melanoma-derived exosomes however reported substantial exosome in the lungs. In two of these studies, B16-BL6 exosomes (murine melanoma) were engineered to express *Gaussia* luciferase (GL exosomes) and streptavidin (SAV-LA exosomes) respectively, showed prominent accumulation in liver and lungs 38, 40. One study demonstrated that a high dose of exosomes administered intravenously resulted in asphyxia as a result of the exosome accumulation in the lungs 19. However, the exosome dose administered in the former two studies (4-5 µg) were much lower than that of the latter (400 µg). Streptavidin was reported to naturally form tetramers in physiological conditions 75, and so the SAV-LA exosomes in the study could have formed aggregates from the interaction between the streptavidin molecules and accumulated in the lungs. However, the authors reported no size differences between the SAV-LA exosomes and unmodified ones from NTA analysis 40. Size analysis however was not performed on the GL exosomes 38. Another study, also using B16BL6 exosomes showed substantial lung accumulation, which was significantly reduced following the disruption of their exosomal surface proteins 72. The surface protein disruption done in this study was by Proteinase K treatment for 30 minutes, which resulted in major ablation of surface proteins on B16BL6 exosomes, compared to the milder disruption on the surface proteins of Exo_B16_ by DTPA incorporation in this study as discussed above. Therefore, the minimal lung accumulation of Exo_B16_ observed in this study could not be attributed to the altered surface proteins. Although both B16BL6 and B16F10 are both melanoma-derived cell lines, the lung metastatic organotropism of the former was reported to be higher than that of the latter 76, thus it is likely that B16BL6 exosomes do home to the lungs to a greater extent than B16F10 exosomes. This is highlighted by a study that demonstrated exosomes derived from cancer cell lines with higher lung metastatic organotropism accumulated in lung tissues 3 times higher than those derived from cell lines with other metastatic organotropisms such as liver, bone and brain 77. Another study showed B16F10 exosomes presence in lungs and bone marrow following intravenous administration, and that they induce greater metastasis of B16F10 cells to these sites compared to untreated controls 78. However, the amount of exosomes present in these tissues were not properly quantified. To complicate the position further, exosome doses administered in these studies were expressed differently (i.e. in terms of particle number or µg protein), which does not allow direct comparison to the results in this work. This implies that the exosome doses administered in these studies may vary and was reported to also influence their biodistribution 33.

Another study by Lai et al. using exosomes from HEK293 cells showed a completely different exosome biodistribution profile to the one presented in this work. HEK293-derived exosomes accumulated to the greatest extent in the kidneys, followed by the liver, lungs and spleen 39. The HEK293 exosomes had their surface engineered to have fusion protein constructs consisting of a PDGF-transmembrane domain for anchoring on the exosomal membrane; a biotin acceptor peptide sequence (BAP) for biotinylation by an exogenously expressed bacterial biotin ligase; and *Gaussia* luciferase. The modified exosomes here also did not show size differences from the unmodified exosomes 39. Again, this study highlights the probable effect of introducing additional moieties of relatively large size on exosomal membrane (e.g. luciferase and streptavidin) on the tissue tropism of exosomes *in vivo*, which concurs with the role of exosomal surface proteins on their tissue localisation discussed above. Substantial considerations are warranted when deciding on the modification status to be adapted for drug delivery applications, and it is therefore imperative that the biodistribution of engineered exosomes be compared with their unmodified counterparts to take into account any possible effect of the modification on their biodistribution, Thus, the membrane radiolabelling approach proposed in this work would serve as an excellent tool for this purpose.

Discrepancies between exosome biodistribution reported in the above studies including the results in this work could also be due to the different labelling and imaging modalities used. Previous work from our group 58, 79 demonstrated the difference between the biodistribution of the PLGA nanocapsules labelled with DiR or radiolabelled with ^111^Indium, where nanocapsules labelled with DiR showed significantly higher lung accumulation as compared to the same nanocapsules which were radiolabelled by including 5-10% PLGA-PEG-DTPA in the excipients during formulation (i.e. without post-synthesis surface modification as done in this current work). Live whole body SPECT/CT imaging showed that the radiolabelled nanocapsules had a substantial lung accumulation at 1 h post-injection, which then continued to decrease over time from 4 h to 24 h, and this was supported by the quantitative organ biodistribution values obtained by gamma counting. Given the non-specific dye exchange phenomenon between membranes associated with lipophilic dyes such as DiR and PKH67 described earlier, the lung accumulation of DiR-labelled nanocapsules observed after 24 h is likely to come from the dye exchanged from the labelled nanocapsules to lung tissue where they initially accumulated in the early timepoint before redistributing to other organs, and this exchanged dye probably accumulated in the lungs over time up to 24 h. This highlights the robustness and reliability of using the nuclear modality in assessing exosome biodistribution and is the main motivation in developing the novel exosome radiolabelling approaches described in this study.

There are reports on naive exosomes having the potential of adopting the homing properties of their parent cell *in vivo* 33 or home to self-tissue *in vitro* 71. In another study, the Exo_B16_ in similar B16F10-bearing C57Bl/6 mice were reported to show tumour accumulation of ~3% total administered fluorescence 33. A separate study using PC3 and MCF-7 exosomes also showed similar self-tissue accumulation of ~2% ID/gT 19. In our hands, fluorescently-labelled B16F10 exosomes (**Scheme S2** - supplementary information) showed a significantly higher uptake than GL261 (murine glioma) exosomes in B16F10 cells *in vitro* **(Fig. S9)**, which suggests the self-homing potential of B16F10 exosomes. However, Exo_B16_ showed very low accumulation in B16F10 tumours *in vivo*, of less than 1% ID/gT. This low tumour accumulation of naïve exosomes, has been attributed to the rapid clearance of exosomes from the circulation by resident macrophages in organs that form part of the reticuloendothelial system (RES). A study demonstrated that depleting macrophages in mice by liposomal clodronate prior to exosome administration significantly increased their circulation time 80. A separate study showed that by blocking the Scavenger Receptor Class A family (SR-A), a recently identified uptake receptor for exosomes in macrophages, liver accumulation of exosomes was significantly reduced while their circulation time increased, which led to a 3-fold increase in tumour accumulation *in vivo* 34. Exosomes expressing CD47, which inhibits phagocytosis by macrophages upon binding to their SIRPα surface protein, were reported to have prolonged circulation time and resulted in better tumour uptake and ablation *in vivo* 81. Flow cytometry analysis on Exo_B16_ showed that CD47 is expressed very minimally on their surface **(Fig. S10)**, which corroborated with the observed rapid clearance of Exo_B16_ from the circulation. Similar analysis done on exosomes derived from other cancer and non-cancer cell lines suggested that CD47 is not a common marker of exosomes, and that this should be taken into account when interpreting the circulation profile of exosomes **(Fig. S10).** This highlights the importance of the endowment of active targeting moieties such as expression of targeting ligands as fusion constructs on exosomal surface proteins 15, 31, as well as developing strategies to bypass the RES organs as described above for effective targeted *in vivo* delivery of exosomes to non-RES sites such as tumours.

Immunodeficient mouse strains of various degrees of immune system impairment are used in studies involving human-derived tumour models to improve the engraftment success in mice. To date, the most immunodeficient mouse strain described is the NOD-*scid ILR2γ^null^* (NSG) mice, whereby the NOD- mutation renders their innate immune cells (particularly macrophages and dendritic cells) defective; the *scid-*mutation results in absence of the adaptive immune cells (T- and B-cells) and complement system, and the complete null mutation of the IL2R gene results in the absence of NK-cells as well as global defective cytokine-dependent signalling 82, 83. In this study, although the B16F10 cells used to develop the subcutaneous tumour is of murine origin and therefore does not require an immunodeficient background for the host, NSG mice were chosen to serve as the extreme counterpart of the immunocompetent C57Bl/6 mice to investigate the influence of the immune system in Exo_B16_ biodistribution. In this study, Exo_B16_ biodistribution did not differ significantly between the C57Bl/6 and the NSG mice. However, looking at the kinetics of Exo_B16_ accumulation in the RES organs of the NSG mice, the spleen initially recorded a lower signal, which increased by 3-fold between 4 h and 24 h to match that of the of the C57Bl6 mice **(Fig. 6C**). This delayed exosome accumulation in RES organs in immunodeficient mice was consistent with a study using NOD.CB17- Prkdcscid/J mice, and was attributed to the defective complement activation in opsonisation and therefore less effective uptake by phagocytic cells in mice with NOD-mutation background 19.

Interestingly, tumours of the immunocompetent mice showed significantly higher accumulation of Exo_B16_ than that of the immunodeficient mice **(Fig. 6D).** Given the difference in the immune background of both mice strains, it is hypothesised that the difference in tumour accumulation values is due to the difference in the proportion of tumour-associated macrophages (TAMs) present in the tumours. Flow cytometry analysis on the total cells isolated from subcutaneous B16F10 tumours (gating strategy is described in supplementary information and **Fig. S11A)** developed in both strains of mice showed that tumours from the immunodeficient NSG mice had a significantly smaller population of TAMs (CD45+ F4/80+ CD11b+) **(Fig. S11B)** compared to that of the tumours from C57Bl/6 mice, which supports the hypothesis above. This suggests that tumour accumulation of exosomes in an immunodeficient host can be an underestimation compared to the actual value in an immunocompetent background. Therefore, it is important to relate the degree of immunity impairment of the animal model used to the results obtained for a more contextual interpretation of the data which might affect factors such as dosing for future therapy studies.

## Conclusions

The results in this work demonstrated that melanoma-derived exosomes were successfully and stably radiolabelled using a novel membrane radiolabelling approach. This has enabled a quantitative analysis of their biodistribution to be carried out in melanoma-bearing immunocompetent mice, showing high accumulation in the liver and spleen from the early time points up to 24 h, with marginal tumour (i.e. self-tissue) accumulation. This membrane radiolabelling approach also enabled a quantitative biodistribution comparison of the same exosomes in a similar tumour model but established in immunodeficient mice and showed that defective immune system did not influence the exosome biodistribution *in vivo* with the exception of the degree of their accumulation in the tumours. This novel membrane radiolabelling method is therefore a simple, reliable and more importantly, has the potential of radiolabelling any type of exosomes, isolated from either primary or immortalised cell cultures, and even from physiological fluids without requiring any engineering on the exosomes. It is hoped that this work will serve as an impetus in achieving a more standardised approach to understanding the *in vivo* fate of the many different types of exosomes thus rendering these future studies more comparable given the heterogeneous nature of exosomes. Given the marginal self-tissue accumulation as opposed to the high RES-organ accumulation of naïve exosomes observed in this study, active targeting moieties appears to be essential to be imparted on exosomes for them to have a better prospect as an effective drug delivery system. Nonetheless, the *in vivo* biodistribution of naïve exosomes should always be studied and compared to that of their engineered counterpart, and this work provides an excellent tool for such comparative studies to be done reliably and accurately.

## Supplementary Material

Supplementary figures and tables.

## Figures and Tables

**Table 1:** Physicochemical characterisation of exosomes

Exosome	Size^1,2^ (nm)	Yield^1,2,3^ (p/ml)	[Protein]^2,4^ (µg/ml)	Particle to protein (P:P) ratio^2,5^ (p/µg)
B16F10	132.3 ± 5.6	2.04x10^13^ ± 3.9x10^12^	451.15 ± 71.5	4.52x10^10^ ± 1.26 x10^10^

^1^Measured using NanoSight LM10. ^2^Values are expressed as mean ± SD, where n=3. ^3^Yield was obtained by cell-conditioned medium pooled from 6 rounds of harvesting from CELLine AD1000 flasks (72 ml). ^4^Measured using BCA assay. ^5^Value obtained by using formula: P:P ratio = Yield / [Protein].
