# Supplementary material for: Membrane Radiolabelling of Exosomes for Comparative Biodistribution Analysis in Immunocompetent and Immunodeficient Mice - A Novel and Universal Approach
Source: Theranostics. 2019 Feb 28;9(6):1666–82. doi: 10.7150/thno.27891 (PMC6485196; doi:10.7150/thno.27891)
Supplement: Supplementary file 1 — Supplementary figures and tables. [file thnov09p1666s1.pdf]

Membrane radiolabelling of exosomes for comparative biodistribution analysis in immunocompetent and immunodeficient mice – a novel and universal approach

Farid N. Faruqu[1], Julie Tzu-Wen Wang[1], Lizhou Xu[1], Luke McNickle[1], Eden Ming-Yiu Chong[1], Adam Walters[1], Mark Gurney[2], Aled Clayton[2], Lesley A. Smyth[3], Robert Hider[1], Jane Sosabowski[4*], Khuloud T. Al-Jamal[1*]

[1] Institute of Pharmaceutical Science, Faculty of Life Sciences & Medicine, King's College London, Franklin-Wilkins Building, 150 Stamford Street, London SE1 9NH, United Kingdom

[2] School of Medicine, Tenovus Building, University Hospital of Wales, Heath Park, Cardiff, CF14 4XN

[3] School of Health Sport and Bioscience, University of East London, Water Lane, London E15 4LZ

[4] Centre for Molecular Oncology, Barts Cancer Institute, Queen Mary University of London, Charterhouse Square, London EC1M 6BQ

*Corresponding authors:
Prof. Khuloud T. Al-Jamal
Email address: khuloud.al-jamal@kcl.ac.uk (K.T. Al-Jamal).

Dr. Jane Sosabowski
Email address: j.k.sosabowski@qmul.ac.uk (J.K. Sosabowski).

Supplementary Materials and Methods

Materials

AlexaFluor® 488 NHS Ester (Succinimidyl Ester) (A20000) was purchased from Thermo Fisher Scientific and dimethyl sulfoxide (10080110) were purchased from Fisher Scientific (UK). Rabbit anti-mouse CD81 (bs-6934R) and CD9 (bs-2489R) antibodies were purchased from Insight Biotechnology (UK). HRP-linked goat anti-rabbit (7074S) was purchased from Cell Signalling Technology (USA). Collagenase Type IV (LS004188) and DNase I (LS002007) were purchased from Worthington. Rat anti-mouse CD45-APC (103111; isotype - 400611), F4/80-FITC (123107; isotype - 400505), CD11b-PE (101207; isotype - 400607), CD47-APC (127513; isotype - 400511) antibodies, and 10X RBC lysis buffer (420301) were purchased from BioLegend (UK). 70 µm cell strainers (352350) were purchased from VWR (UK).

Cell culture

B16F10 and GL261 cells were both cultured in Advanced RPMI medium supplemented with 10% exosome-depleted FBS, 1% GlutaMax, and 1% Penicillin-Streptomycin in an incubator maintained at 37°C and 5% CO_2.

Fluorescent labelling of exosomes

AlexaFluor® 488 NHS Ester (Alexa-NHS) was used to fluorescently label lysine (Lys) residues on the surface of B16F10 exosomes (Exo_{B16}). Alexa-NHS was reacted with Exo_{B16} at a 1:400 molar ratio (Lys:Alexa-NHS – it was assumed that 1 exosome is equivalent to 1 BSA molecule i.e. containing 59 lysine residues) protected from light for 1 hour at room temperature (RT). Labelled Exo_{B16} (Alexa- Exo_{B16}) was purified from excess unreacted Alexa-NHS using Sepharose® CL-2B columns (self-packed according to the dimensions of the commercially available NAP-5™ columns) optimised such that exosomes elute in the first 2 x 500 µl fractions.

Exosomal surface proteins detection by dot blot following mock radiolabelling

Exo_{B16} were subjected to a mock membrane-radiolabelling protocol (as described in the Methods section of the main manuscript, but without the addition of $^{111}InCl_3$ to the 0.2 M ammonium acetate buffer pH 5.5). 40 µl of 5 x 10^{10} p/ml unlabelled and labelled

Exo_{B16} in PBS was spotted onto a nitrocellulose membrane (10 µl at a time) and dried under a nitrogen stream. Non-specific binding on the membrane was blocked by 3% milk in Tris-buffered saline with 0.1% Tween-20 (TBS-T, pH 7.6) at RT for 1 hour. The membrane was incubated with primary antibodies (1:1000 dilution) in 3% milk in TBS-T (i.e. the blocking buffer) at 4°C overnight. Washing was done 3 times with TBS-T for 5 minutes each. The membrane was then incubated with HRP-conjugated rabbit anti-mouse secondary antibody (1:1000 dilution) in the blocking buffer for 1 hour at RT. Washing was done 3 times with TBS-T for 5 minutes each as above. The washed membrane was incubated with the ECL substrate for 3 min, and then imaged using the Gel Doc™ system (Bio-Rad, US) under the “Intense Bands” setting. The acquired imaged was processed using Image Lab™ software (Bio-Rad, US).

***In vitro* uptake of exosomes in cancer cell lines**

B16F10 and GL261 cells were seeded in 96-well plates at a density of 30,000 cells per well and left to settle overnight. Each cell line was then treated with 2.5 x 10^{10} Alexa- Exo_{B16} for 1, 4, and 24 hours. Penicillin-Streptomycin (10% of total volume in each well) was added to prevent bacterial growth. After treatment, cells were detached, resuspended in 3% FBS/PBS and run on FACSCalibur under the FL1 channel for detection of AlexaFluor® 488 signals. The results were analysed using CellQuest Pro software (BD Biosciences, US). Untreated B16F10 and GL261 cells were used as control.

Tumour-associated macrophages (TAMs) population analysis

Female C57Bl/6 mice and male NOD SCID gamma (NSG) mice (~20 g, 6-8 weeks old) were inoculated with B16F10 cells (1 x 10^6 cells in 100 µl PBS) subcutaneously into the left and right rear flanks of the mice to establish subcutaneous (SC) B16F10 tumours. The mice were monitored closely post-inoculation and were culled when the tumours reached ~200-300 mm^3. The tumours were excised, and each tumour was chopped into fine pieces in 1 ml serum-free media, which was then topped up to 4 ml with serum-free media. The following enzymes were added to the tumour suspension at their respective final concentrations: Collagenase Type IV (2 mg/ml) and DNase I (150 µg/ml). The tumours were digested for 45 minutes at 37°C in a shaking water bath (320 rpm), vortexing every 15 minutes. The reaction was stopped by adding 10

ml 1% BSA solution (prepared in PBS), and the cell suspension was passed through a 70 µm cell strainer. Tumour-derived cells in the filtrate were washed once with PBS and subjected to a red blood cell lysis step by adding 5 ml 1X RBC lysis buffer to the cell pellet post-washing and incubated for 5 minutes at RT. The reaction was stopped by adding 25 ml PBS. Cells were pelleted (500 g, 5 min) and resuspended in 400 µl PBS. Cells were single- and triple-stained with rat anti-mouse CD45-APC, F4/80-FITC and CD11b-PE antibodies (and their respective isotype controls; 1:200 dilution in 100 µl PBS) for 20 min at 4°C. Cells were washed once with PBS and run on FACSCalibur under FL1, FL2 and FL4 channels with the appropriate compensation, and the results were analysed using FlowJo software (FlowJo LLC, US).

Supplementary figures

A

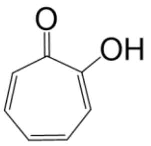

B

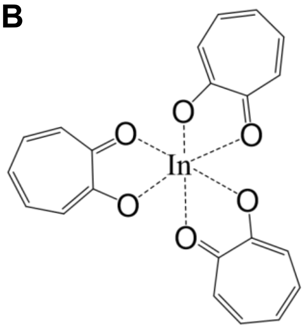

C

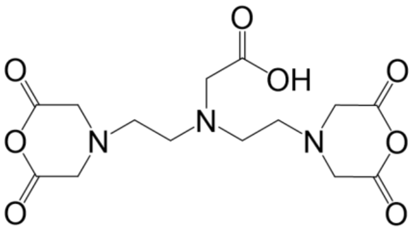

D

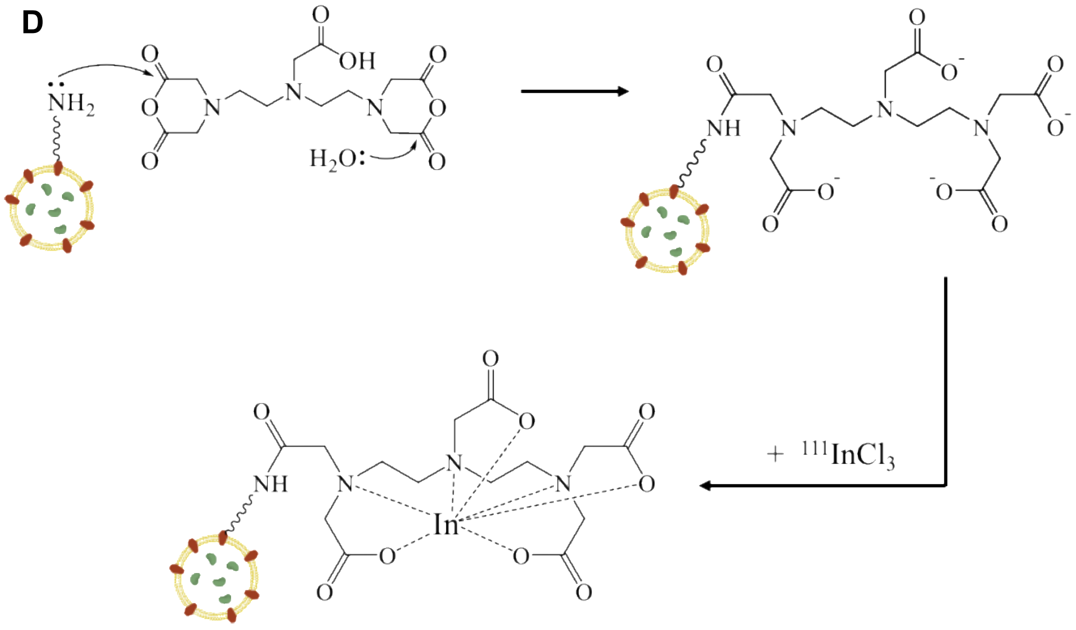

Scheme S1 Chemical structure and reactions of key molecules in this study. (A) Chemical structure of a tropolone molecule. **(B)** 3 tropolone molecules coordinating a single $^{111}In^{3+}$ ion in aqueous solution, forming the [^{111}In]Trop complex. **(C)** Chemical structure of a cyclic DTPA-dianhydride molecule. **(D)** Conjugation of DTPA-anhydride to exosomes by formation of a stable amide bond and subsequent radiolabelling by chelating $^{111}In^{3+}$.

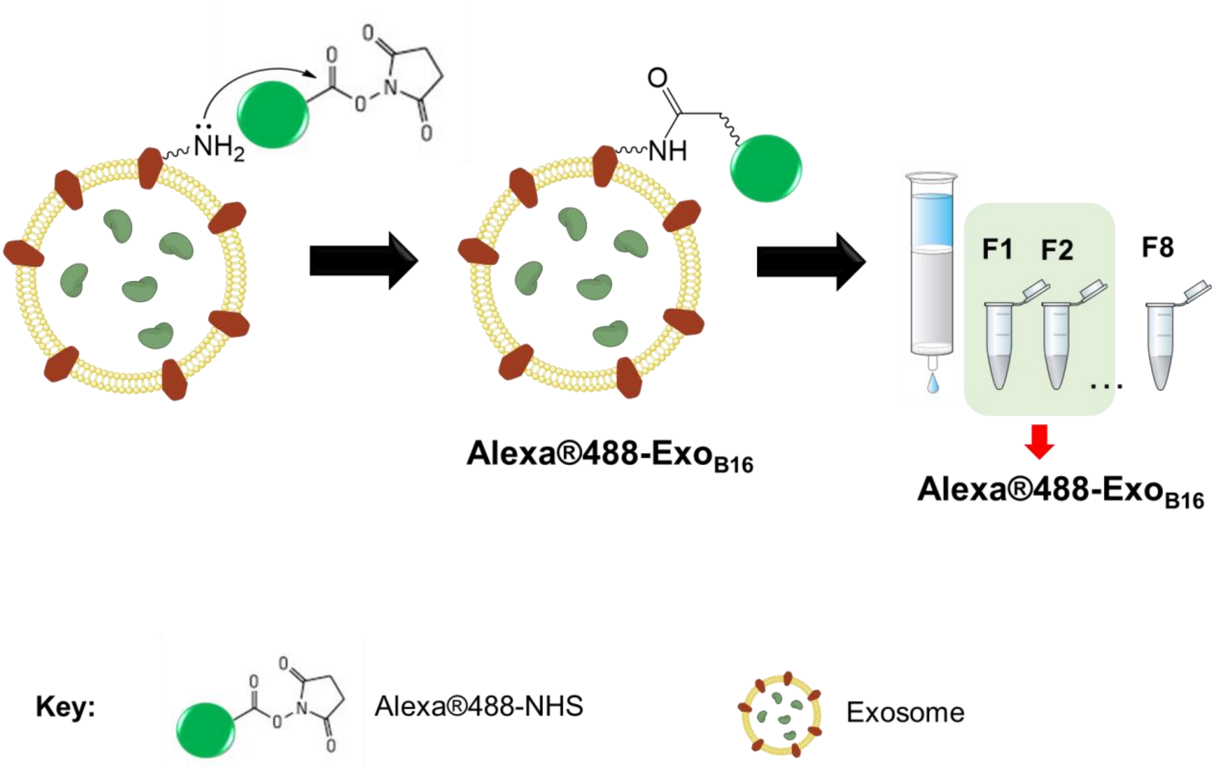

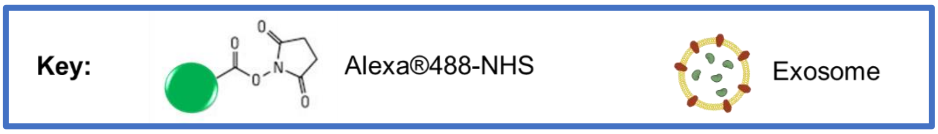

Scheme S2 Fluorescent labelling of exosomes with Alexa®488-NHS

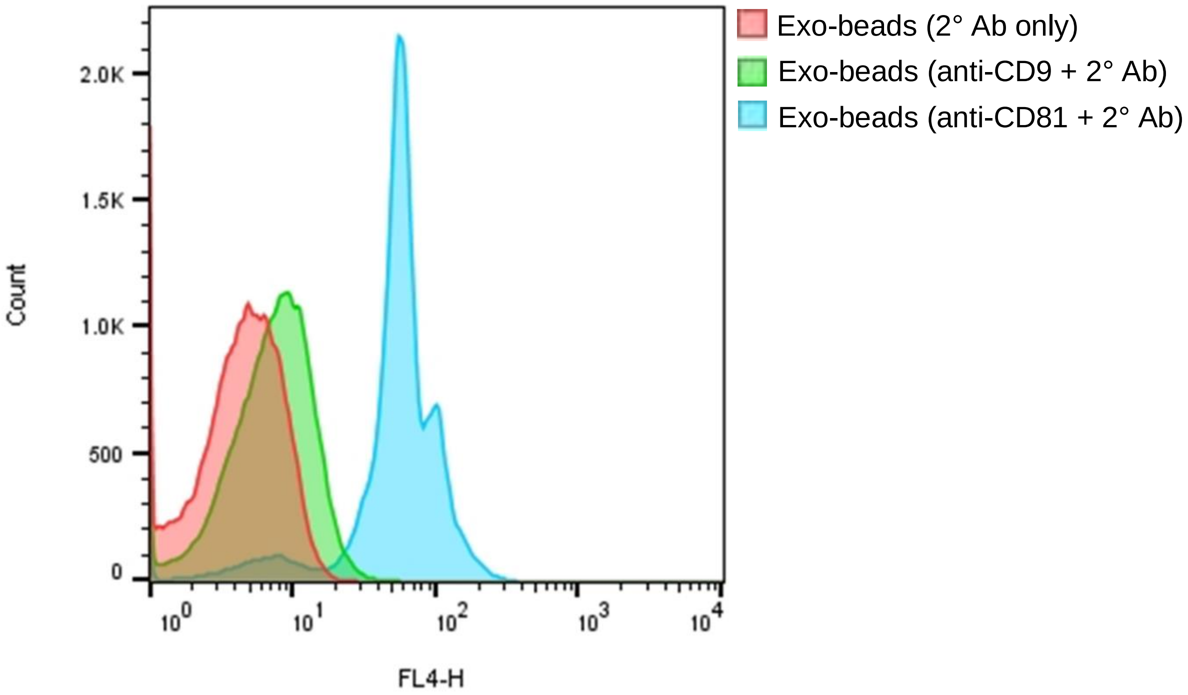

Fig. S1 Biochemical analysis of B16F10 exosomes. Histograms illustrate the detection of CD81 and CD9 using flow cytometry on exosomes isolated from B16F10 cells. Exosomes were coupled to aldehyde/sulphate latex beads prior to detection. Exo-beads complex were subsequently stained using a 2-step labelling (anti-CD81 or anti-CD9 1° ab/Cy5-conjugated 2° ab). Exo-beads complex stained with Cy5-conjugated 2° ab only was used as control. The shift in FL4 signals from the control indicates positive expression of the markers on the exosomes.

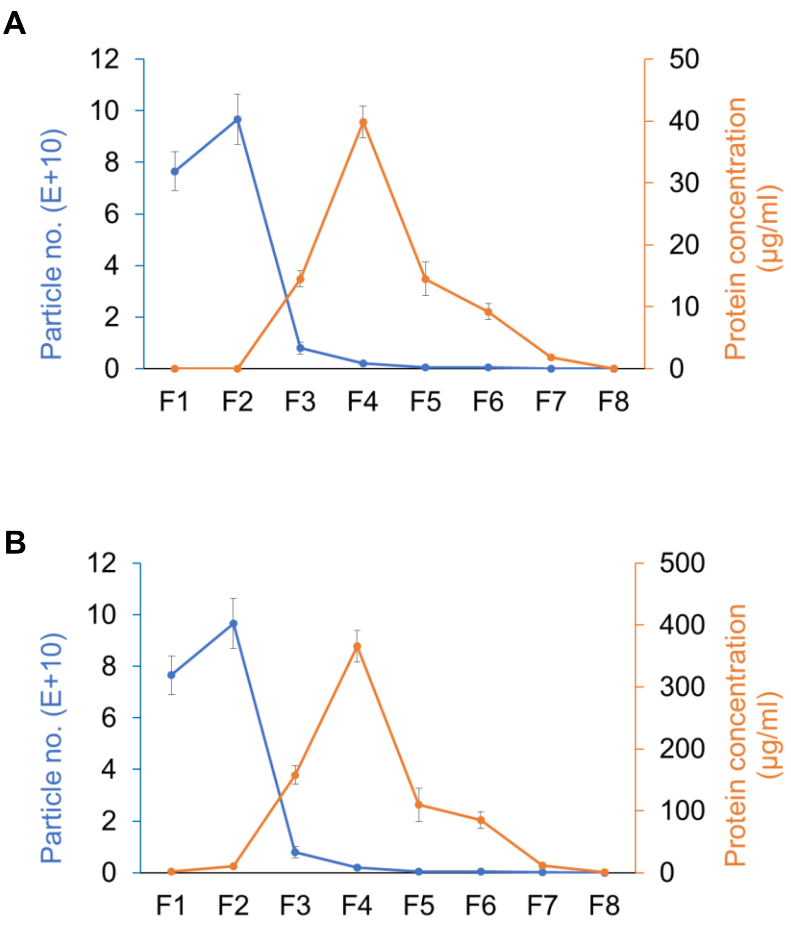

Fig. S2 Exosome and protein elution profile by gel filtration. B16F10 exosomes and BSA solution were loaded separately onto Sepharose® CL-2B columns self-packed according to the dimensions of the commercially available NAP-5 columns. 3.5 x 10^{11} exosomes (100 µl) were loaded onto the column. **(A)** 80 µg/ml and **(B)** 800 µg/ml BSA solution was loaded onto the column (100 µl for both concentrations). Eight 500 µl fractions were collected and the particle number and protein concentration in each fraction was measured using Nanosight and microBCA assay respectively. Values are expressed as mean ± SD, where n=3.

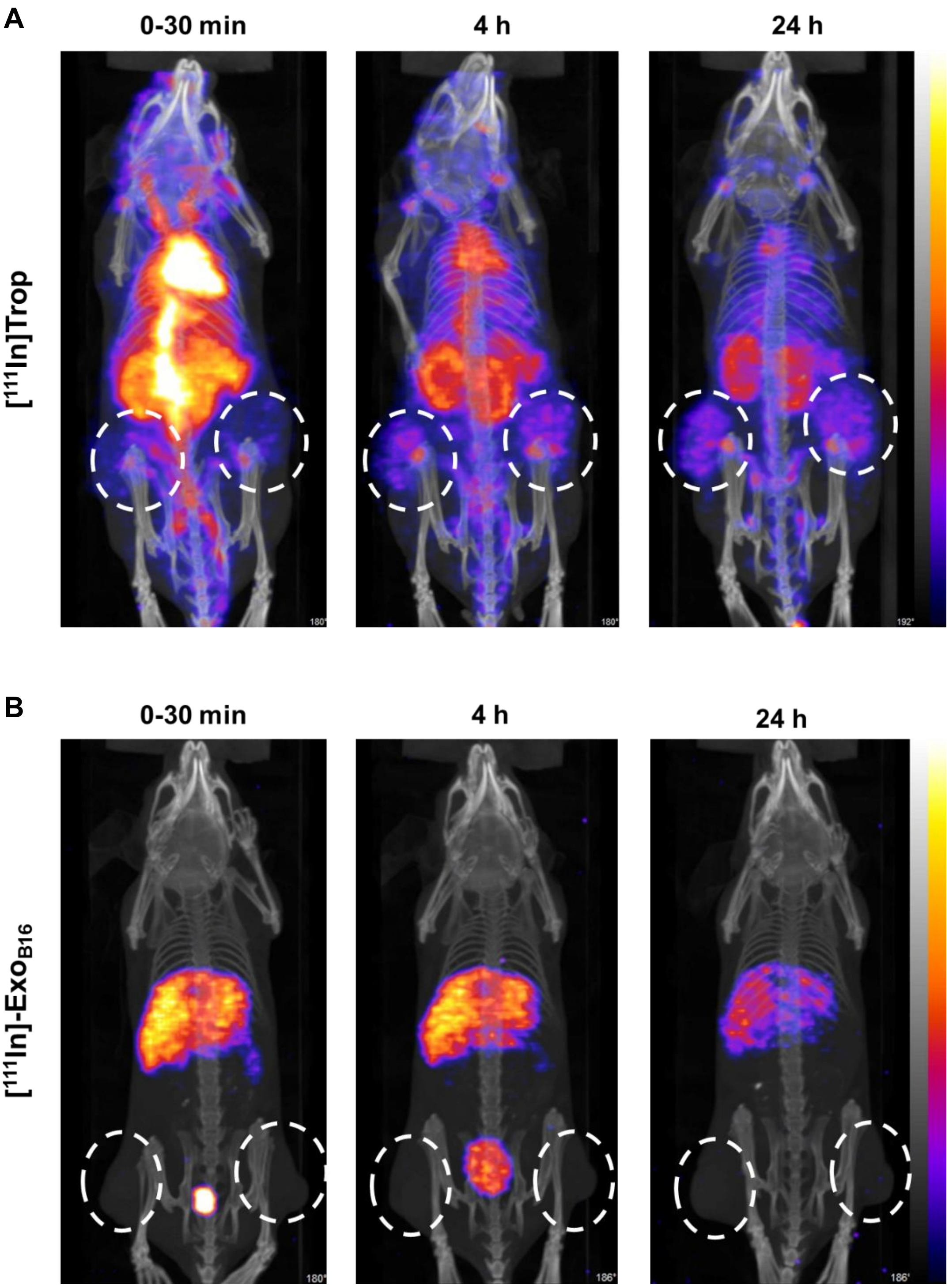

Fig. S3 Whole body SPECT/CT imaging of intraluminal-labelled B16F10 exosomes in subcutaneous B16F10 tumour-bearing C57Bl/6 mice. (A) Mice were injected intravenously with just [^{111}In]Trop complex as control. **(B)** Mice were injected with [^{111}In]-Exo$_{B16}$. Imaging was done immediately, 4, and 24 h post-injection. White circles indicate the position of tumours.

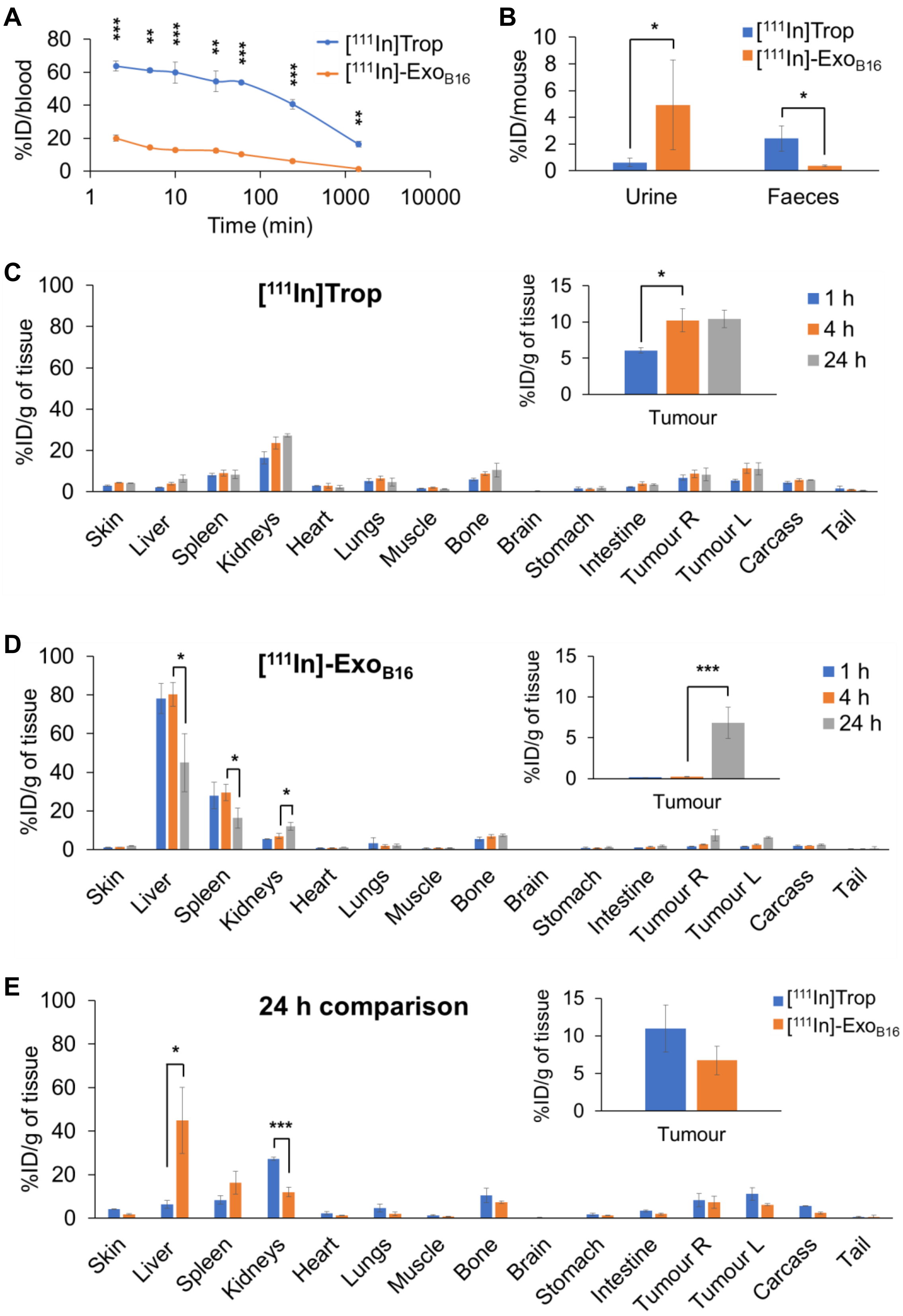

Fig. S4 Blood circulation, excretion and organ biodistribution profiles of intraluminal-labelled B16F10 exosomes in melanoma-bearing C57Bl/6 mice. The [^{111}In]Trop group was injected with 2 µg tropolone complexed with 0.5-1MBq of $^{111}InCl_3$ while the [^{111}In]-Exo$_{B16}$ group was injected with 1x10^{11} [^{111}In]-Exo$_{B16}$ (0.5-1MBq). **(A)** Blood circulation profile of [^{111}In]Trop and [^{111}In]-Exo$_{B16}$. 5 µl blood were taken *via* tail bleeding at

2 min, 5 min, 10 min, 30 min, 1 h, 4 h and 24 h following intravenous injection of each compound. **(B)** Excretion profile of [^{111}In]Trop and [^{111}In]-Exo$_{B16}$ where urine and faeces were collected from the animals 24 h post-injection. **(C)** and **(D)** Organ biodistribution of [^{111}In]Trop and [^{111}In]-Exo$_{B16}$ respectively. Animals were culled at 1 h, 4 h and 24 h post-injection, perfused with saline and their organs were excised for analysis by gamma counting. Inset shows the zoomed-in tumour accumulation values for each group. **(E)** Comparison of organ biodistribution of [^{111}In]Trop and [^{111}In]-Exo$_{B16}$ 24 h post-injection, where inset shows zoomed-in tumour accumulation values for each group. Values are normalised to organ weight and expressed as mean ± SD, where n=3 for each group. For **(C), (D)** and **(E)**, statistical analyses were done on liver, spleen, kidneys and tumour (p*<0.05, p** < 0.01, p*** <0.001).

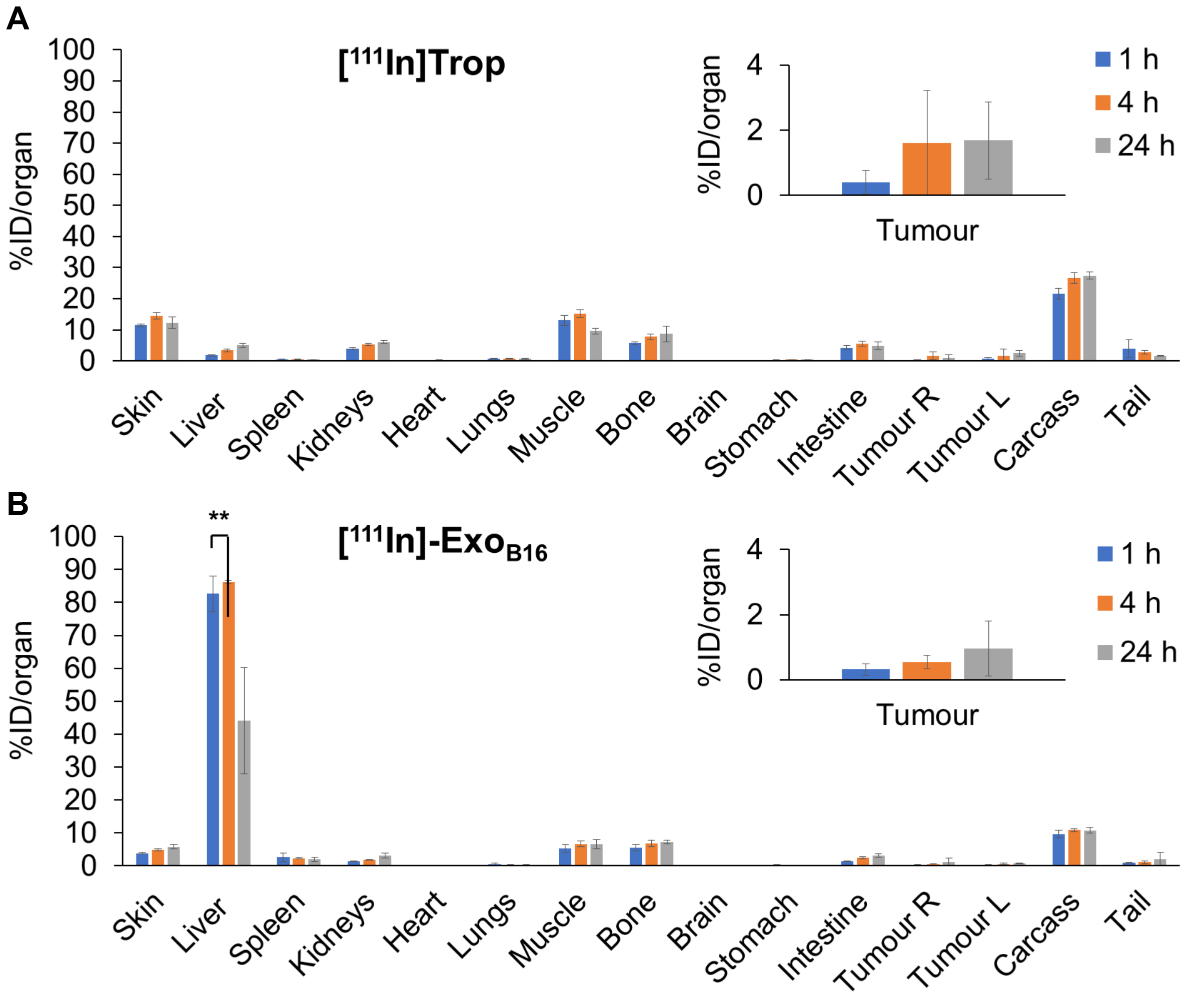

Fig. S5 Blood circulation, excretion and organ biodistribution profiles of intraluminal-labelled B16F10 exosomes in subcutaneous B16F10 tumour-bearing C57Bl/6 mice. The [^{111}In]Trop group was injected with 2 µg tropolone complexed with 0.5-1MBq of $^{111}InCl_3$ while the [^{111}In]-Exo$_{B16}$ group was injected with 1x10^{11} [^{111}In]-Exo$_{B16}$ (0.5-1MBq). **(A)** and **(B)** Organ biodistribution of [^{111}In]Trop and [^{111}In]-Exo$_{B16}$ respectively. Animals were culled at 1 h, 4 h and 24 h post-injection, perfused with saline and their organs were excised for analysis by gamma counting. Inset shows the zoomed-in tumour accumulation values for each group. Values are expressed as mean ± SD, where n=3 for each group. For **(C), (D)** and **(E)**, statistical analyses were done on liver, spleen, kidneys and tumour ($p^{**}<0.01$).

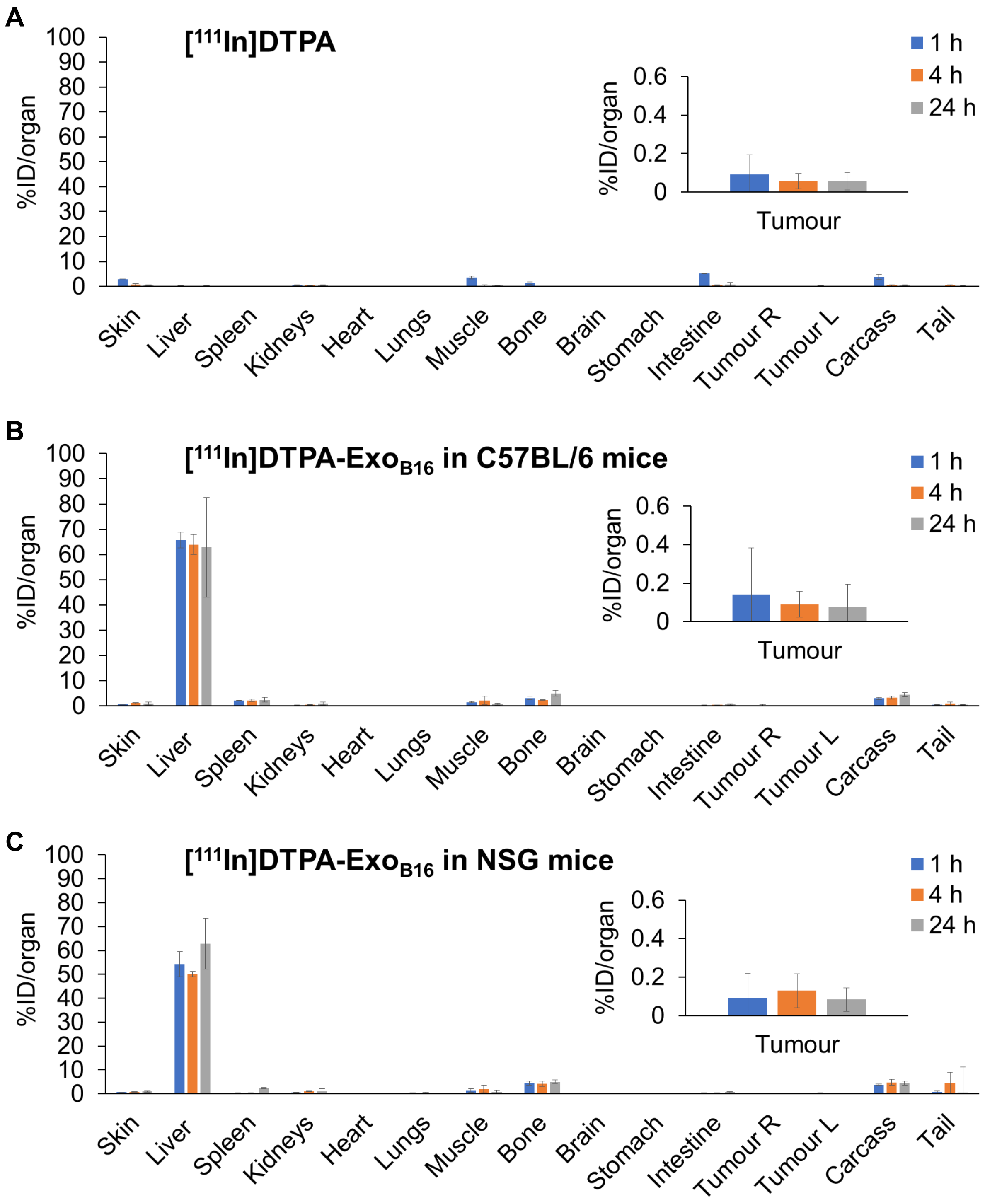

Fig. S6 Blood circulation, excretion and organ biodistribution profiles of membrane-labelled B16F10 exosomes in subcutaneous B16F10 tumour-bearing mice. The $[^{111}In]$DTPA group was injected with 0.02M DTPA complexed with 0.5-1MBq of $^{111}InCl_3$ while the $[^{111}In]$DTPA-Exo_{B16} group was injected with $1x10^{11}$ $[^{111}In]$DTPA-Exo_{B16} (0.5-1MBq). **(A)** and **(B)** Organ biodistribution of $[^{111}In]$DTPA and $[^{111}In]$DTPA-Exo_{B16} respectively in C57Bl/6 mice. **(C)** Organ biodistribution of $[^{111}In]$DTPA-Exo_{B16} in NSG mice. Animals were culled at 1 h, 4 h and 24 h post-injection, perfused with saline and their organs were excised for analysis by gamma counting. Inset shows the zoomed-in tumour accumulation values for each group. Values are expressed as mean ± SD, where n=3 for each group. Statistical analyses were done on liver, spleen, kidneys and tumour (p*<0.05, p** < 0.01, p*** <0.005).

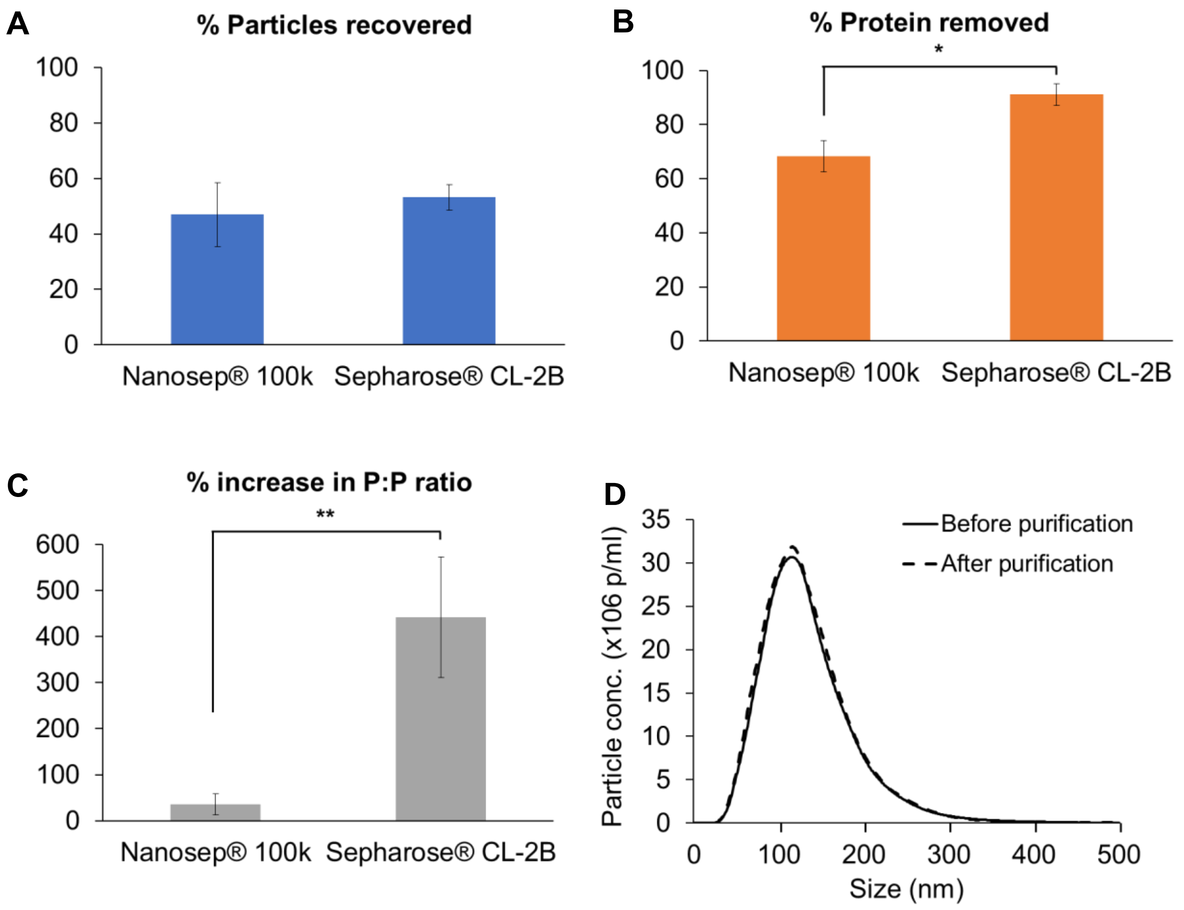

Fig. S7 Assessment of particle recovery and improvement in purity of exosome samples following an additional purification step prior to radiolabelling. Two different purification methods were compared – ultrafiltration using Nanosep®100k MWCO; and gel filtration using Sepharose® CL-2B**.** About 1 x 10^{10} exosomes (100 µl) were used as the initial amount. Sepharose® CL-2B columns were self-packed according to the dimensions of the commercially available NAP-5™ columns. **(A)** Comparison of the percentage of particles recovered post-purification. **(B)** Comparison of the percentage of contaminating proteins eliminated post-purification. **(C)** Comparison of the improvement in purity of exosome sample expressed as the particle:protein ratio (P:P). Values are expressed as mean ± SD, where n=3, and Student's t-test was used for statistical analysis (p*<0.05, p** < 0.01). **(D)** Histograms shows the size distribution of exosomes before and after purification through the Sepharose® CL-2B column, measured using NanoSight LM10.

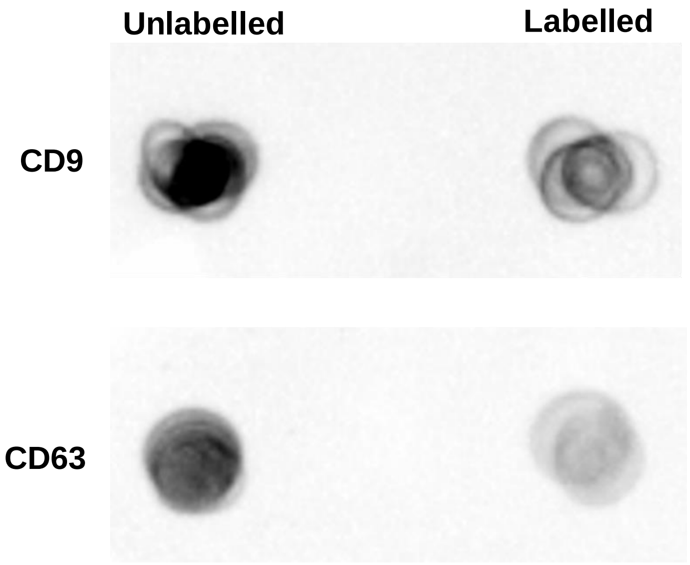

Fig. S8 Exosomal surface proteins detection by dot blot following mock membrane-radiolabelling. Exo_{B16} were subjected to a mock membrane-radiolabelling protocol (as described in the Methods section of the manuscript, but without the addition of $^{111}InCl_3$ to the 0.2 M ammonium acetate buffer). Equal numbers (40 µl from 5 x 10^{10} p/ml stock) of both unlabelled and labelled of Exo_{B16} were then blotted on a nitrocellulose membrane, followed by blocking in 3% milk solution. The membrane is then incubated with primary rabbit anti-mouse CD9 and CD63 antibodies, followed by incubation with HRP-linked goat anti-rabbit secondary antibodies. After addition of the ECL substrate, the membrane was imaged using Gel Doc™ system (Bio-Rad, USA) and the image processed using Image Lab™ software (Bio-Rad, USA).

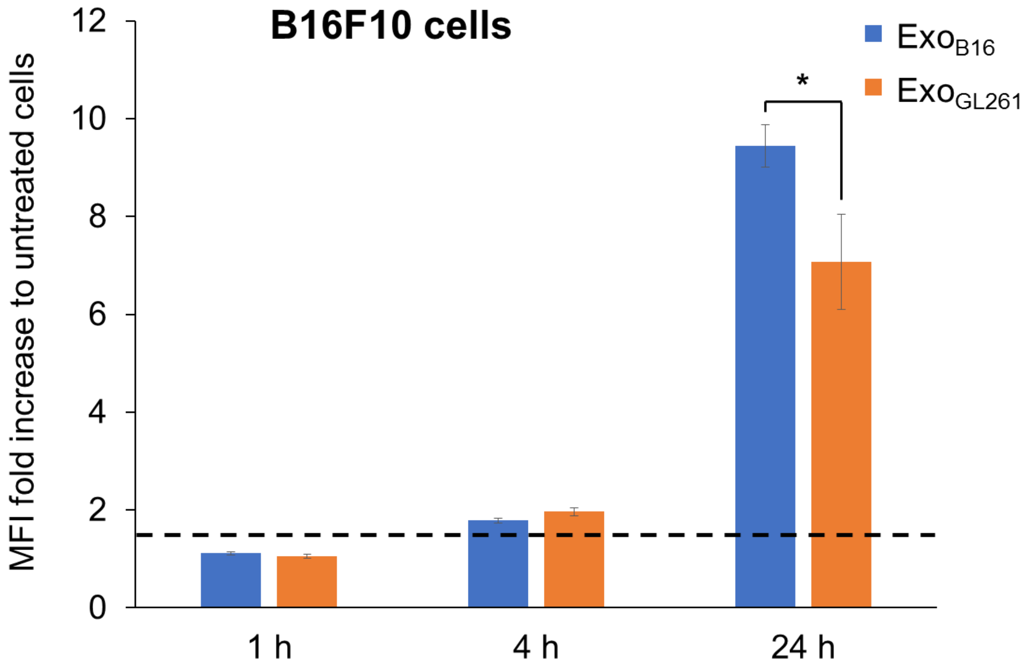

Fig. S9 Uptake of fluorescently-labelled exosomes in B16F10 cells *in vitro*. B16F10- (Exo_{B16}) and GL261-derived exosomes (Exo_{GL261} – murine glioma) were fluorescently labelled with AlexaFluor®488-NHS dye. B16F10 cells were then incubated with 2.5 x 10^{10} fluorescently-labelled Exo_{B16} and Exo_{GL261} for 1, 4 and 24 h, after which the cells were collected and analysed using flow cytometry under the FL1 channel. Degree of exosome uptake are expressed as the fold difference in median fluorescence intensity (MFI) from that of untreated cells. MFI fold difference value of at least 1.5 (dashed line) is regarded as uptake in the B16F10 cells. Values are expressed as mean ± SD, where n=3, and Student's t-test was used for statistical analysis ($p^* < 0.05$).

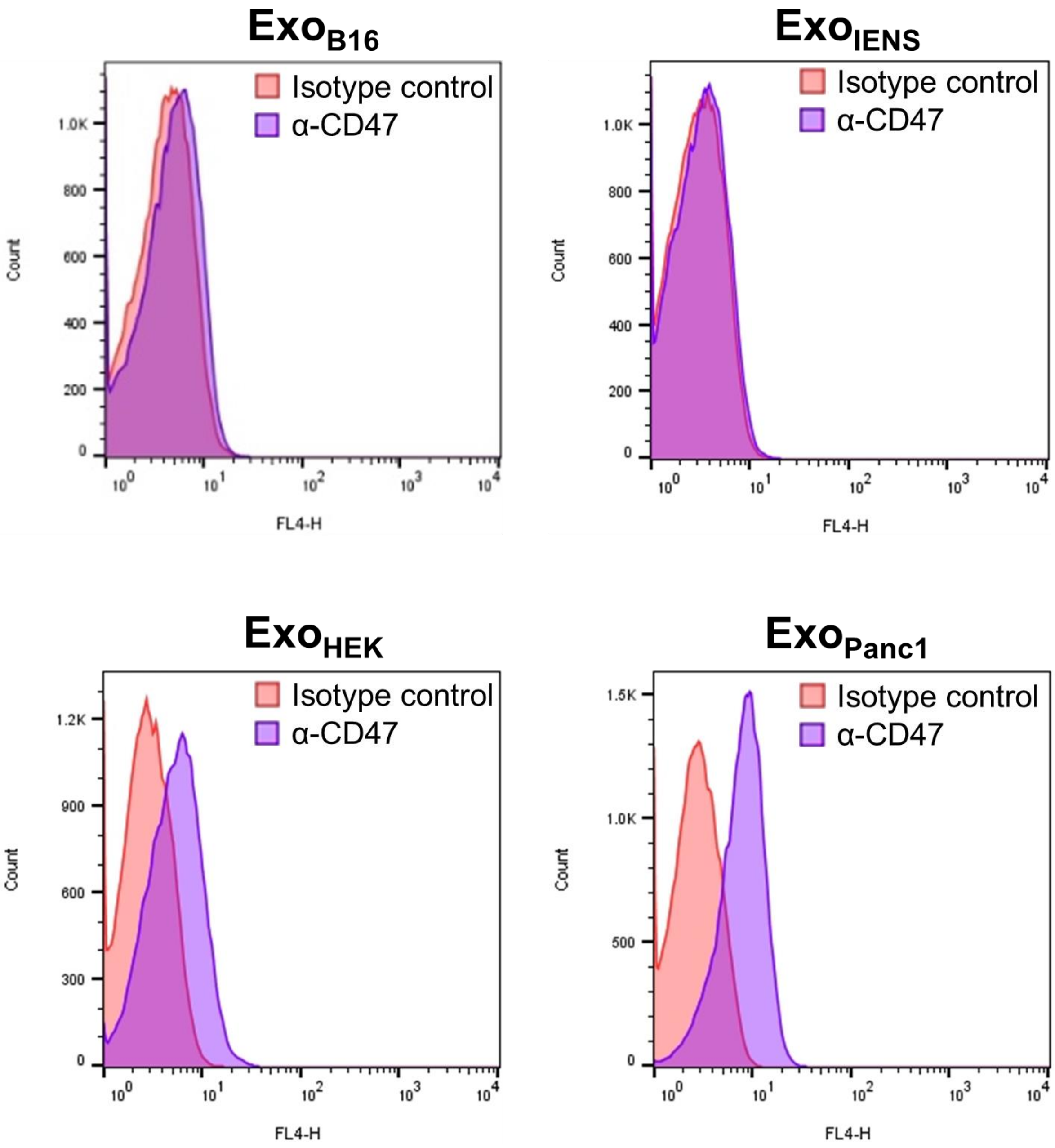

Fig. S10 CD47 expression on exosomes derived from cancer and non-cancer cell lines. Histograms illustrate the detection of CD47 on exosomes isolated from B16F10 (Exo_{B16} – murine melanoma), IENS (Exo_{IENS} – murine glioblastoma stem cells), HEK293 (Exo_{HEK} - human epithelial) and Panc1 (Exo_{Panc1} – human pancreatic adenocarcinoma) cell lines using flow cytometry. Exosomes were coupled to aldehyde/sulphate latex beads prior to detection. Exo-beads complex were then stained with APC-conjugated anti-CD47 or their respective isotype controls (rat anti-mouse for murine exosomes, or mouse anti-human for human exosomes). The shift in FL4 signals indicates positive expression of CD47 on the exosomes.

A

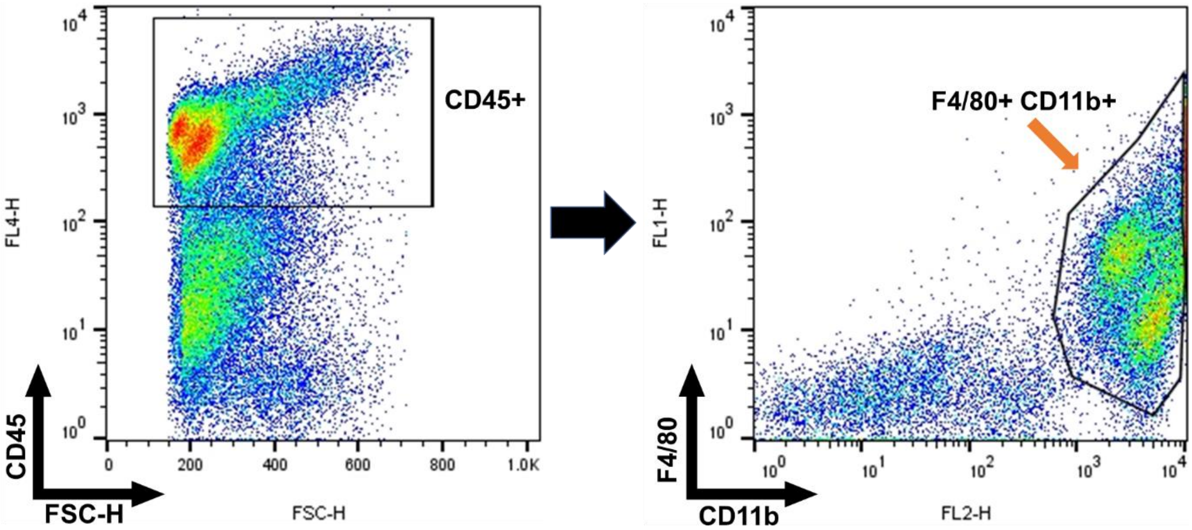

B

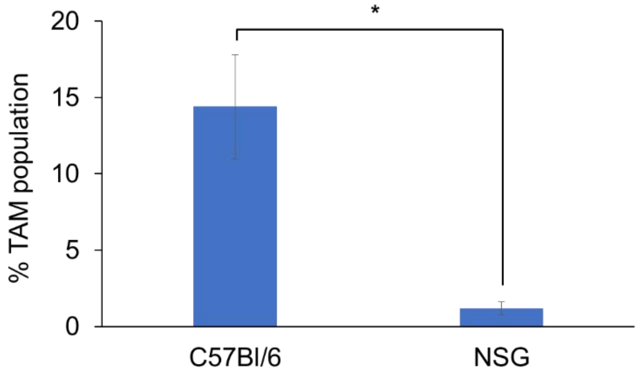

Fig. S11 Comparison of tumour-associated macrophages (TAMs) population between tumours developed in immunocompetent and immunodeficient mice. (A) Representative gating strategy used to identify TAM population among cells isolated from tumours developed in both C57Bl/6 and NSG mice. Cells were triple-stained with APC-conjugated anti-CD45, FITC-conjugated anti-F4/80 and PE conjugated anti-CD11b antibodies. TAMs are identified as CD45+ F4/80+ CD11b+. First, viable cells which are CD45+ are selected, followed by cells which are both F4/80+ CD11b+. **(B)** Population of TAM isolated from tumours developed in C57Bl/6 and NSG mice, expressed as the percentage of CD45+ F4/80+ CD11b+ cells in total cells isolated from the tumours. Values are expressed as mean ± SD, where n=3, and Student's t-test was used for statistical analysis ($p^* < 0.05$).
